# Integrated analysis of tRNA-derived small RNAs in proliferative human aortic smooth muscle cells

**DOI:** 10.1186/s11658-022-00346-4

**Published:** 2022-06-15

**Authors:** Jian-Zhi Zhao, Qi-Yao Li, Jia-Jie Lin, Li-Yun Yang, Mei-Yang Du, Yu Wang, Ke-Xin Liu, Ze-An Jiang, Huan-Huan Li, Si-Fan Wang, Bo Sun, Shi-Qing Mu, Bin Li, Kun Liu, Miao Gong, Shao-Guang Sun

**Affiliations:** 1 Department of Biochemistry and Molecular Biology, Key Laboratory of Medical Biotechnology of Hebei Province, Cardiovascular Medical Science Center, Hebei Medical University, Shijiazhuang, China; 2 Department of Laboratory Medicine, Nanfang Hospital, Southern Medical University, Guangzhou, China; 3 Department of Emergency Surgery, The Second Hospital of Hebei Medical University, Shijiazhuang, China

**Keywords:** tsRNA, VSMC, proliferation, p53, MFN2

## Abstract

**Background:**

Abnormal proliferation of vascular smooth muscle cells (VSMCs) contributes to vascular remodeling diseases. Recently, it has been discovered that tRNA-derived small RNAs (tsRNAs), a new type of noncoding RNAs, are related to the proliferation and migration of VSMCs. tsRNAs regulate target gene expression through miRNA-like functions. This study aims to explore the potential of tsRNAs in human aortic smooth muscle cell (HASMC) proliferation.

**Methods:**

High-throughput sequencing was performed to analyze the tsRNA expression profile of proliferative and quiescent HASMCs. Quantitative real-time polymerase chain reaction (qRT-PCR) was performed to validate the sequence results and subcellular distribution of AS-tDR-001370, AS-tDR-000067, AS-tDR-009512, and AS-tDR-000076. Based on the microRNA-like functions of tsRNAs, we predicted target promoters and mRNAs and constructed tsRNA–promoter and tsRNA–mRNA interaction networks. Gene Ontology (GO) and Kyoto Encyclopedia of Genes and Genomes (KEGG) pathway analyses were performed to reveal the function of target genes. EdU incorporation assay, Western blot, and dual-luciferase reporter gene assay were utilized to detect the effects of tsRNAs on HASMC proliferation.

**Results:**

Compared with quiescent HASMCs, there were 1838 differentially expressed tsRNAs in proliferative HASMCs, including 887 with increased expression (fold change > 2, *p* < 0.05) and 951 with decreased expression (fold change < ½, *p* < 0.05). AS-tDR-001370, AS-tDR-000067, AS-tDR-009512, and AS-tDR-000076 were increased in proliferative HASMCs and were mainly located in the nucleus. Bioinformatics analysis suggested that the four tsRNAs involved a variety of GO terms and pathways related to VSMC proliferation. AS-tDR-000067 promoted HASMC proliferation by suppressing p53 transcription in a promoter-targeted manner. AS-tDR-000076 accelerated HASMC proliferation by attenuating mitofusin 2 (MFN2) levels in a 3′-untranslated region (UTR)-targeted manner.

**Conclusions:**

During HASMC proliferation, the expression levels of many tsRNAs are altered. AS-tDR-000067 and AS-tDR-000076 act as new factors promoting VSMC proliferation.

**Supplementary Information:**

The online version contains supplementary material available at 10.1186/s11658-022-00346-4.

## Background

The death toll of cardiovascular diseases increases year by year, among which ischemic heart disease and cerebrovascular disease account for more than 80% of deaths [1]. Vascular smooth muscle cells (VSMCs), one of the cell types in blood vessels, mainly contract and relax to maintain blood pressure and flow [2]. Abnormal proliferation of VSMCs is a critical process in the pathogenesis of numerous vascular remodeling diseases, such as atherosclerosis [3], hypertension [4], vascular stenosis [5], and diabetic vascular complications [6]. Therefore, exploration of VSMC proliferation can contribute to the early diagnosis and treatment of pathological vascular conditions.

With the development of deep sequencing technology, tsRNAs, a new class of small noncoding RNAs derived from tRNAs, have been discovered in various organisms [7]. Interestingly, the roles of tsRNAs in mediating cardiovascular diseases by regulating VSMC proliferation have been gradually revealed recently [8–10]. For example, tRF^GlnCTG^ and tRF-Gly-GCC promote VSMC proliferation [8, 9], whereas 5′-tiRNA-Cys-GCA inhibits VSMC proliferation, migration, and dedifferentiation by reducing the levels of its target gene signal transducer and activator of transcription 4 (STAT4) [10]. However, the actual repertoire of tsRNAs affecting VSMC proliferation remains unclear.

This study aims to explore the roles of tsRNAs in VSMC proliferation. AS-tDR-000067 (tRFdb_ID: 3003a) targeted the promoter of p53 gene, and AS-tDR-000067 suppression inhibited HASMC proliferation by augmenting p53 levels. AS-tDR-000076 (tRFdb_ID: 3008a) targeted the 3′-UTR of MFN2 mRNA, and AS-tDR-000076 silencing limited HASMC proliferation by enhancing MFN2 levels. This study innovatively documents meaningful tsRNAs associated with HASMC proliferation and highlights AS-tDR-000067 and AS-tDR-000076 as promising factors that promote VSMC proliferation.

## Methods

### Cell culture

Proliferative HASMCs (ScienCell, CA, USA) were induced by smooth muscle cell medium (SMCM, ScienCell) containing 2% fetal bovine serum (FBS, ScienCell), 1% 100× smooth muscle cell growth supplement (SMCGS, ScienCell), and 1% 100× penicillin/streptomycin solution (ScienCell). Quiescent HASMCs were stimulated by FBS and SMCGS starvation for 24 h. Cells were cultured at 37 °C in an incubator containing 5% CO_2_.

### tsRNA-seq library preparation and sequencing

To screen tsRNAs related to HASMC proliferation, we performed high-throughput RNA sequencing of three sets of proliferative and quiescent HASMCs. Total RNA of proliferative and quiescent HASMCs was extracted using an RNA simple total RNA kit (TIANGEN, Beijing, China). Total RNA samples were pretreated to eliminate the interference of RNA modification in the construction of small RNA-seq libraries: (1) 3-aminoacyl (charged) was deacylated to 3′-OH for 3′ adaptor ligation; (2) 3′-cP (2′,3′-cyclic phosphate) was removed to 3′-OH for 3′ adaptor ligation; (3) 5′-OH (hydroxyl group) was phosphorylated to 5′-P for 5′-adaptor ligation; (4) m1A and m3C were demethylated for effective reverse transcription. Subsequently, tsRNA-seq libraries were constructed using a commercial kit for tsRNA sequencing library preparation (Illumina, CA, USA). The kit includes 3′-adapter and 5′-adapter ligation, complementary DNA (cDNA) synthesis, and library PCR amplification. PCR-amplified fragments with a size of 135–160 bp (corresponding to the size range of 15–40 nt small RNA) were selected as tsRNA-seq libraries. Finally, the prepared tsRNA-seq libraries were quantified using an Agilent 2100 Bioanalyzer (Agilent Technologies, CA, USA) and then sequenced using an Illumina NextSeq 500 (Illumina). Sequencing was performed by AKsomics (Shanghai, China). The tsRNA nomenclature used in this paper is derived from AKsomics, where AS indicates the abbreviation of AKsomics, tDR stands for tRNA-derived small RNA, and the number represents the order in which the tsRNA was found during sequencing. In addition, in our sequencing data, we also present the tRFdb_IDs of tsRNAs documented in the tRFdb database [11].

### Sequencing data analysis

Image analysis and base calling were performed by Solexa pipeline v1.8 (Off-Line Base Caller software, v1.8). Valid sequences were preserved by alignment statistical analysis for subsequent tsRNA expression profile analysis and differential expression analysis. Sequencing quality was tested by FastQC software, and NovoAlign software (v2.07.11) was applied to align the trimmed reads (with 5′, 3′-adaptor bases removed) with the mature tRNA and pre-tRNA sequences of GtRNAdbb: Genomic tRNA Database (http://gtrnadb.ucsc.edu/). Remaining reads were aligned to the transcriptome including mRNA/rRNA/snRNA/piRNA/snoRNA/miRNA biotypes. The expression profile and differentially expressed tsRNAs (DEtsRNAs) were calculated based on standardized transcripts per million (TPM). DEtsRNAs can be obtained through GEO series accession number GSE164540 (https://www.ncbi.nlm.nih.gov/geo/query/acc.cgi?acc=GSE164540).

### Quantitative real-time polymerase chain reaction (qRT-PCR)

Total small RNAs (smRNAs) of proliferative and quiescent HASMCs were isolated using a MiRcute miRNA Isolation Kit (TIANGEN). The quantity and integrity of total smRNAs were measured using NanoDrop ND-1000 (Thermo Fisher Science, Massachusetts, USA) and 1% agarose gel electrophoresis. Total smRNAs were reverse-transcribed using a miRcute Plus miRNA First-Strand cDNA Kit (TIANGEN). qRT-PCR was performed using SYBR Green analysis in the miRcute Plus miRNA qPCR Kit (TIANGEN). The reaction conditions for all samples were initial denaturation at 95 °C for 10 min, followed by 40 cycles of heat denaturation at 95 °C for 10 s, annealing at 60 °C for 20 s, and extension at 72 °C for 10 s. All samples were normalized using U6 as internal control, and the 2^−∆∆Ct^ method was applied to calculate the fold change of expression of tsRNAs. The primers are presented in Additional file 1. The experiment was repeated three times for each gene.

### Nuclear and cytoplasmic RNA detection

Nuclear and cytoplasmic components of proliferative HASMCs were isolated using a Nuclear/Cytosol Fractionation Kit (BioVision, CA, USA) following the manufacturer’s instructions. Extraction, quantification, and integrity detection of smRNAs were consistent with the above. The reaction conditions for qRT-PCR were in keeping with the above. GAPDH and U6 were applied as positive controls for the cytoplasm and nucleus, respectively. The primers are listed in Additional file 1. The experiment was repeated three times.

### Construction of tsRNA–promoter interaction networks

Based on the seed region (tsRNA nucleotides 2–8), downstream target promoters of four DEtsRNAs (AS-tDR-001370, AS-tDR-000067, AS-tDR-009512, and AS-tDR-000076) were predicted by RNAhybrid [12] and MiRanda [13]. To further screen HASMC proliferation-related genes in a promoter-targeting manner, Venn analysis was performed between predicted genes containing target promoters and differentially expressed mRNAs (DEmRNAs) in proliferative HASMCs (GSE77279) (fold change > 2 or < ½, *p* < 0.05) [14]. Target genes enriched in GO terms or pathways related to VSMC proliferation were selected to construct the tsRNA–promoter interaction networks using Cytoscape_v3.7.1. All target genes containing target promoters are listed in Additional file 2.

### Construction of tsRNA–mRNA interaction networks

Based on the seed sequence targeting the 3′-untranslated region (UTR) of mRNA, downstream target genes of four DEtsRNAs (AS-tDR-001370, AS-tDR-000067, AS-tDR-009512, and AS-tDR-000076) were predicted by TargetScan [15] and MiRanda [13]. Venn analysis between predicted target genes and DEmRNAs in the proliferative HASMCs (GSE77279) (fold change < 2/3, *p* < 0.05) [14] was performed to obtain target DEmRNAs. Target genes enriched in GO terms or pathways related to VSMC proliferation were selected to construct the tsRNA–mRNA interaction networks utilizing Cytoscape_v3.7.1. In addition, based on the seed sequence targeting the 3′-UTR of mRNAs, downstream target genes of AS-tDR-013295 and AS-tDR-001583 were predicted by TargetScan [15]. Venn analysis between predicted target genes and DEmRNAs in the proliferative HASMCs (GSE77279) (fold change > 1.5, *p* < 0.05) [14] was performed to obtain target DEmRNAs. The tsRNA–mRNA interaction networks were constructed utilizing Cytoscape_v3.7.1. All target DEmRNAs are listed in Additional file 3.

### GO and pathway analyses

For the GO and pathway analyses of target DEmRNAs and HASMC proliferation-related genes containing target promoters, we explored the potential function of AS-tDR-001370, AS-tDR-000067, AS-tDR-009512, and AS-tDR-000076 on HASMC proliferation using the DAVID database [16].

### Antisense oligonucleotide transfection

Antisense oligonucleotides (ASOs) of AS-tDR-000067, AS-tDR-000076, and negative control (ASO-Control) were designed and synthesized by GENEWIZ (Beijing, China). According to the instructions, Lipofectamine 3000 (Invitrogen, CA, USA) was used to transfect ASO-Control, ASO-AS-tDR-000067, or ASO-AS-tDR-000076 into HASMCs. After 7 h, the transfection medium was replaced with fresh SMCM containing 2% FBS, 1% 100× SMCGS, and 1% 100× penicillin/streptomycin solution and cultured to the appropriate time point. Sequences of related ASOs are listed in Additional file 4.

### Western blot analysis

Total protein in HASMCs was extracted using radioimmunoprecipitation assay (RIPA) buffer (Solarbio, Beijing, China) and 1 mM phenylmethylsulfonyl fluoride (PMSF; Solarbio). Sodium dodecyl sulfate (SDS)-polyacrylamide gel electrophoresis (PAGE) was performed to separate equal amounts of proteins and transfer them onto the polyvinylidene fluoride (PVDF) membrane (Merck, Darmstadt, Germany). After blocking with 5% skimmed milk at 37 °C for 2 h, the membranes were incubated overnight at 4 °C with the following specific primary antibodies: p53 (1:1000; Wanleibio, Shenyang, China), mitofusin 2 (MFN2, 1:500; Abcam), α-smooth muscle actin (α-SMA, 1:500; Wanleibio), proliferating cell nuclear antigen (PCNA, 1:1000, Proteintech, Wuhan, China), β-actin (1: 1,000, Proteintech), and glyceraldehyde-3-phosphate dehydrogenase (GAPDH, 1:1000; Wanleibio). After washing three times in Tris-buffered saline within Tween 20 (TBST) for 5 min, the membranes were incubated in goat anti-rabbit secondary antibody (dilution at 1:20,000, Sino Biological) for 1 h at room temperature. A ChemiDoc™ MP Imaging System (BIO-RAD, CA, USA) was employed to visualize the protein signals and quantify band strength. The experiment was repeated at least three times.

### Cell proliferation analysis

After the corresponding treatment of HASMCs, a 5-ethynyl-2′-deoxyuridine (EdU) incorporation assay was performed according to the instructions of the BeyoClick™ EdU Cell Proliferation Kit and Alexa Fluor 594 (Beyotime, Shanghai, China). EdU-positive cells were observed by fluorescence microscope (Olympus IX71, Tokyo, Japan). The experiment was repeated three times.

### Dual-luciferase reporter gene assay

Dual-luciferase reporter assays were performed to evaluate the direct binding of AS-tDR-000067 to the p53 promoter, as well as AS-tDR-000076 to the MFN2 3′-UTR. Using GPtransfect-Mate (GenePharma, Shanghai, China), luciferase reporter gene vector (psi-CHECKTM-2 Vector, Promega, USA) containing the wild-type (WT) p53 promoter was cotransfected into 293A cells with AS-tDR-000067 mimic or AS-tDR-000067 mutant (GenePharma). Similarly, the vector containing the WT MFN2 3′-UTR was cotransfected with AS-tDR-000076 mimic or AS-tDR-000076 mutant (GenePharma). After 24 h, the Dual-Luciferase® Reporter Assay System (Promega) was performed based on the manufacturer’s instructions. For each analysis, the *Renilla* luciferase signal was normalized to the firefly luciferase signal. Sequences of tsRNA mimics and tsRNA mutant are listed in Additional file 5. The experiment was repeated three times.

### Statistical analysis

All data are from at least three independent experiments, expressed as mean ± standard deviation (SD). Student’s *t*-test was performed to compare the differences between two groups. *p* < 0.05 was considered statistically significant.

## Results

### Expression profile analysis and qRT-PCR detection of tsRNAs

**Fig. 1:**
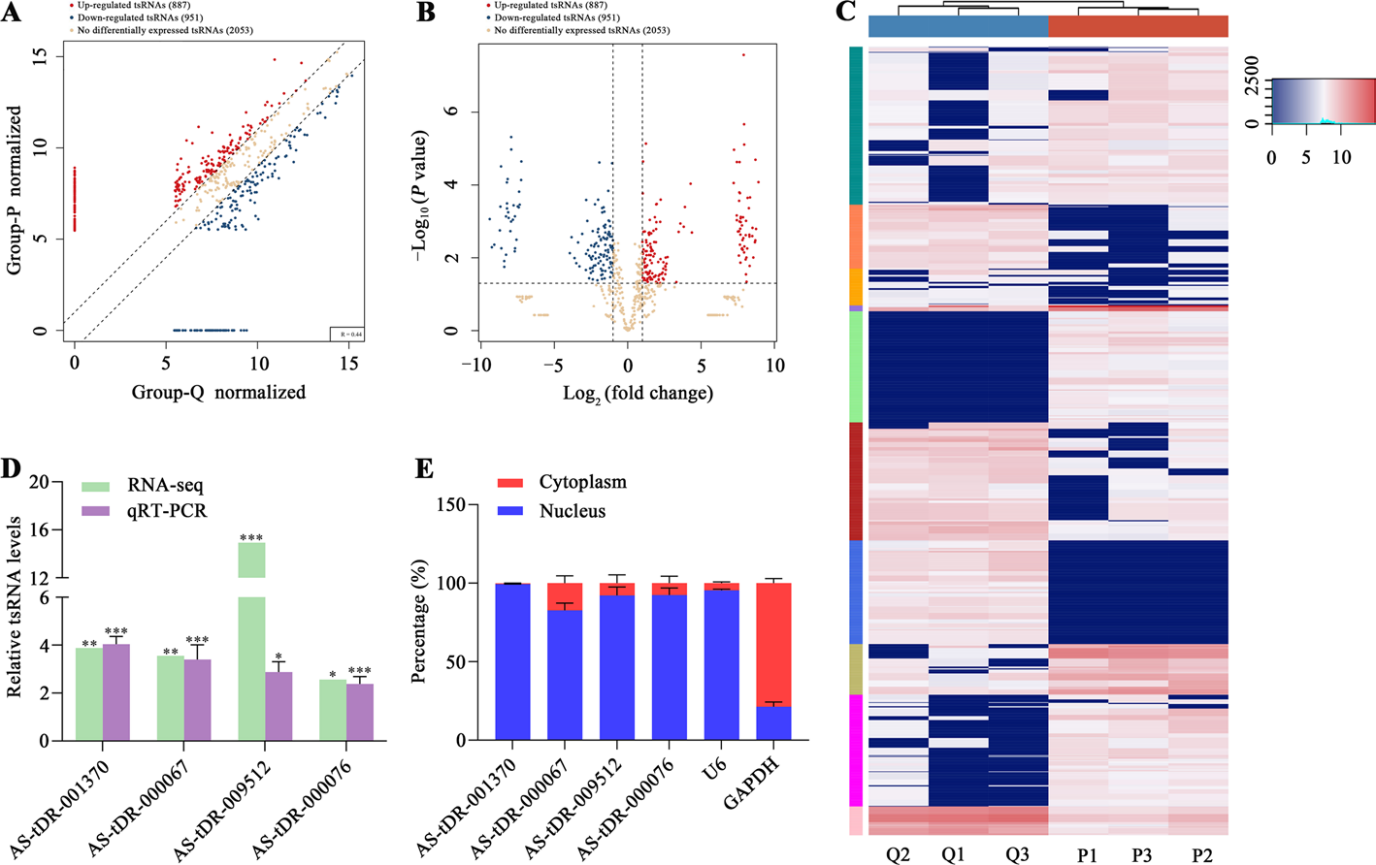
Expression profile analysis and qRT-PCR detection of tsRNAs. **A** Scatter plot of DEtsRNAs between proliferative and quiescent HASMCs. TPM values (log_2_ scaled) of all tsRNAs; **B** Volcano plot of DEtsRNAs between proliferative and quiescent HASMCs; **C** Heatmap of DEtsRNAs between proliferative and quiescent HASMCs. Each row represents a tRNA, while each column represents a sample. P1, P2, and P3 represent three samples of proliferative HASMCs; Q1, Q2, and Q3 represent three samples of quiescent HASMCs; **D** Expression of four DEtsRNAs in proliferative and quiescent HASMCs was detected by qRT-PCR and normalized to U6. Data presented as mean ± SD of three independent experiments, *p < 0.05, **p < 0.01, ***p < 0.001 versus quiescent HASMCs. The *p*-value represents the possibility of a difference in tsRNA expression between proliferative and quiescent HASMCs; **E** AS-tDR-001370, AS-tDR-000067, AS-tDR-009512, and AS-tDR-000076 were abundant in the nucleus of HASMCs. U6 and GAPDH acted as positive controls in the nucleus and cytoplasm, respectively. Data shown as mean ± SD of three independent experiments

Compared with quiescent HASMCs, PCNA levels were increased together with decreased α-SMA levels, and cell proliferation rate was significantly augmented in proliferative HASMCs (Additional file 6), suggesting successful induction of two cell phenotypes for screening HASMC proliferation-related tsRNAs. Using RNA sequencing, we screened 3891 tsRNAs to explore their expression profiles in two HASMC phenotypes. The scatter plot in Fig. 1A, volcano plot in Fig. 1B, and hierarchical clustering in Fig. 1C illustrate that, in contrast to quiescent HASMCs, proliferative HASMCs possessed 887 increased DEtsRNAs and 951 decreased DEtsRNAs (fold change > 2 or < ½, *p* < 0.05). According to the fold change and expression abundance, four DEtsRNAs (AS-tDR-001370, AS-tDR-000067, AS-tDR-009512, and AS-tDR-000076) were selected for qRT-PCR detection. Consistent with the sequencing, AS-tDR-001370, AS-tDR-000067, AS-tDR-009512, and AS-tDR-000076 were higher in proliferative HASMCs (Fig. 1D). Nuclear/cytoplasmic RNA detection revealed that the four DEtsRNAs were predominantly localized in the nucleus of HASMCs (Fig. 1E).

### Construction of tsRNA–promoter interaction networks

**Fig. 2:**
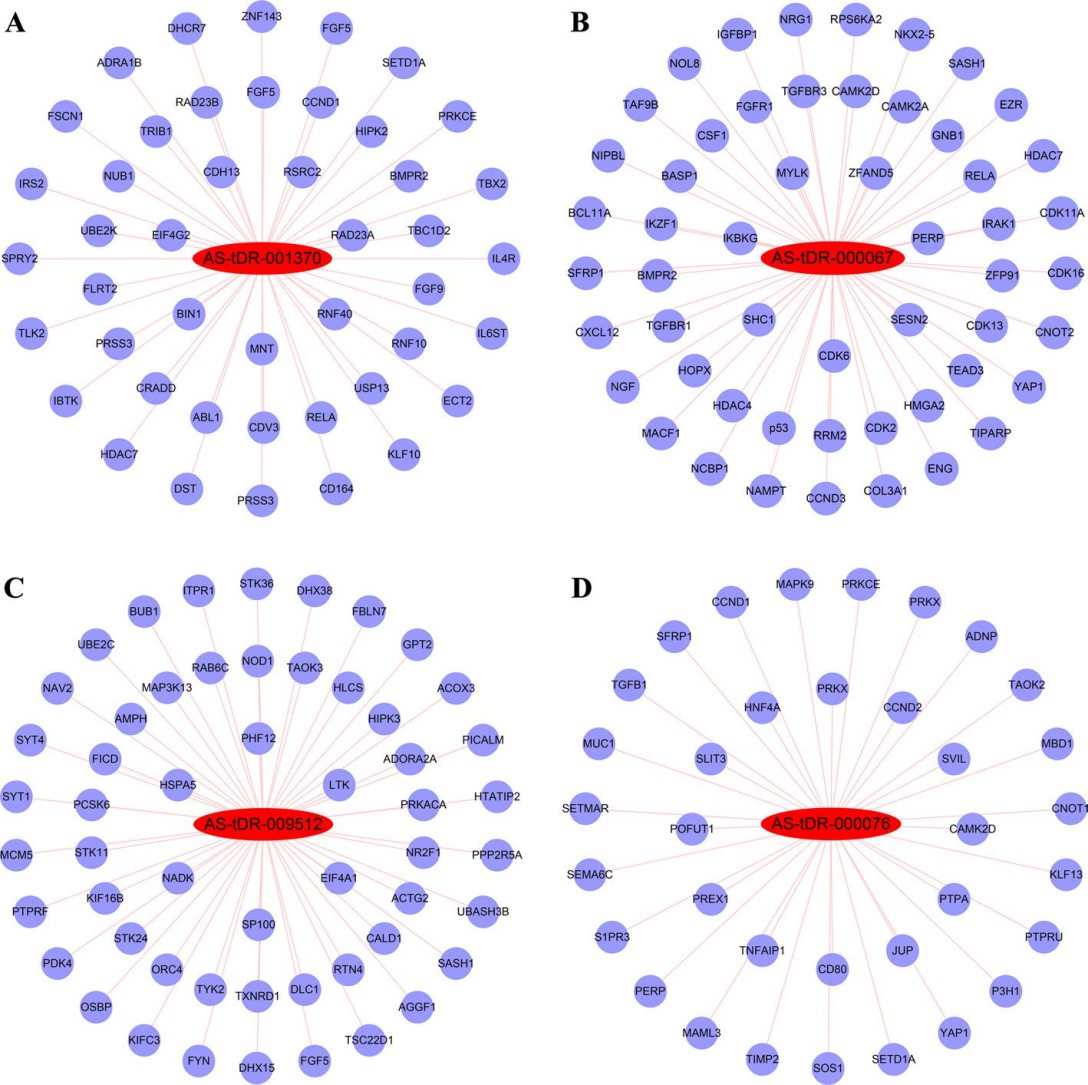
Construction of tsRNA–promoter interaction networks. Subnetworks of **A** AS-tDR-001370, **B** AS-tDR-000067, **C** AS-tDR-009512, and **D** AS-tDR-000076. Red nodes indicate increased DEtsRNAs, while blue nodes indicate proliferation-related genes containing target promoters

The functions of tsRNAs binding to Argonaute (AGO) proteins are similar to miRNAs [17]. The binding of nuclear miRNAs to promoters leads to activation [18] or silencing [19] of the transcription of target genes. We thus speculated that tsRNAs are similar to miRNAs and play regulatory roles by targeting promoters. Four DEtsRNAs (AS-tDR-001370, AS-tDR-000067, AS-tDR-009512, and AS-tDR-000076) were selected to construct tsRNA–promoter interaction networks (Fig. 2).

### Functional annotation of genes containing target promoters

GO and pathway analyses of genes containing target promoters were performed to explore the potential of AS-tDR-001370, AS-tDR-000067, AS-tDR-009512, and AS-tDR-000076 in HASMC proliferation using the DAVID database [15]. GO analysis revealed that the four DEtsRNAs were enriched in histone deacetylase binding (ontology: molecular function, GO: 0042826), peptidyl-threonine phosphorylation (ontology: biological process, GO:0018107), hippo signaling (ontology: biological process, GO: 0035329), regulation of stem cell population maintenance (ontology: biological process, GO: 2000036), regulation of cell cycle arrest (ontology: biological process, GO: 0071156), and negative regulation of cell growth (ontology: biological process, GO: 0030308) (Additional file 7). In addition, pathway analysis revealed that the four DEtsRNAs were enriched in mitogen-activated protein kinase (MAPK) signaling pathway (hsa04010), p53 signaling pathway (hsa04115), vascular smooth muscle contraction (hsa04270), Wnt signaling pathway (hsa04310), and other cell proliferation-related pathways (Additional file 7).

### Construction of tsRNA–mRNA interaction networks

**Fig. 3:**
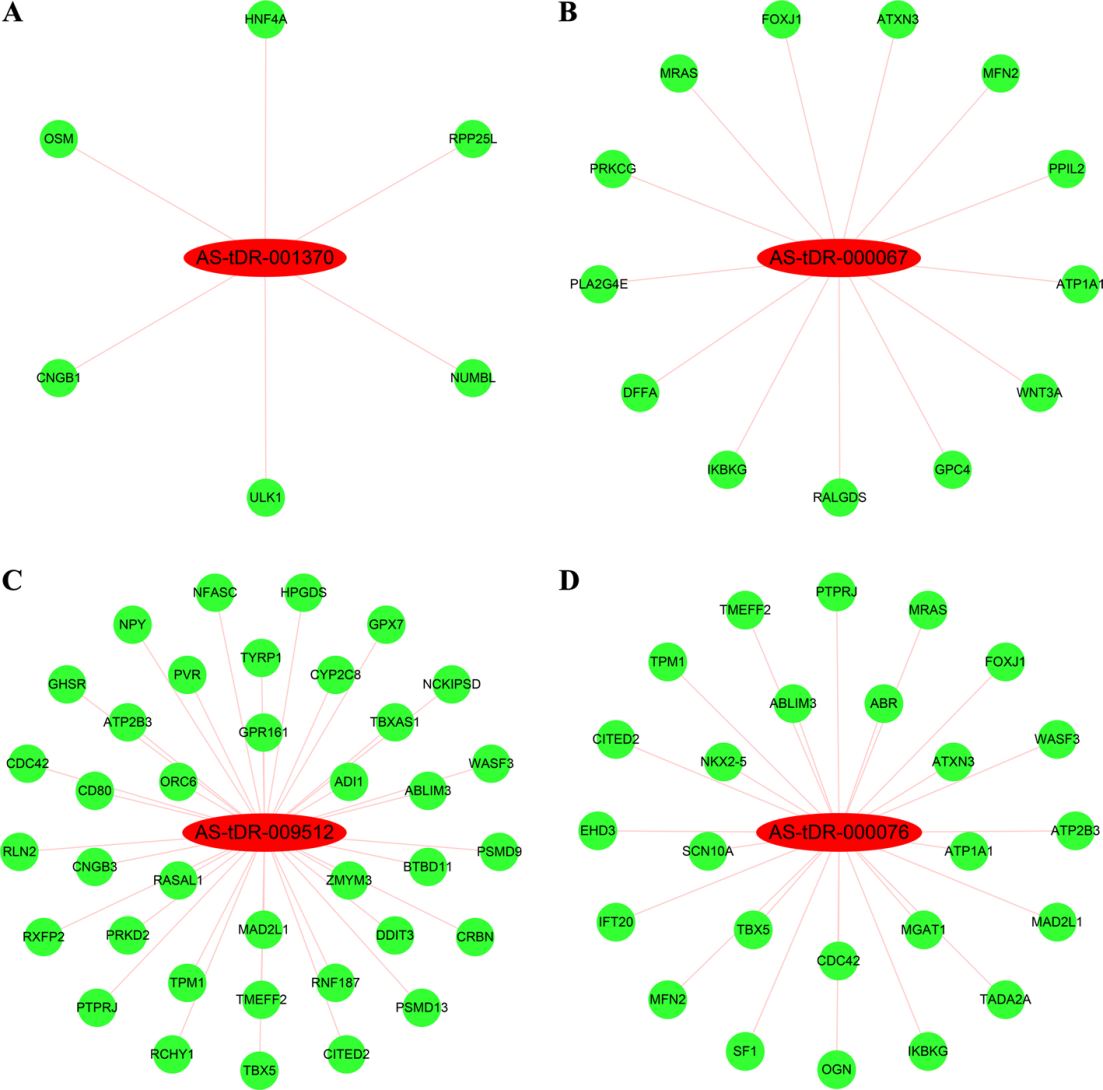
Construction of tsRNA–mRNA interaction networks. Subnetworks of **A** AS-tDR-001370, **B** AS-tDR-000067, **C** AS-tDR-009512, and **D** AS-tDR-000076. Red nodes indicate increased DEtsRNAs, while green nodes indicate proliferation-related decreased target DEmRNAs

Similar to miRNAs, tsRNAs inhibit mRNA expression in a sequence-dependent manner by binding to AGO [20]. Four DEtsRNAs (AS-tDR-001370, AS-tDR-000067, AS-tDR-009512, and AS-tDR-000076) were selected to construct tsRNA–mRNA interaction networks (Fig. 3).

### Functional annotation of target DEmRNAs

GO and pathway analyses of target DEmRNAs were performed to explore the potential of AS-tDR-001370, AS-tDR-000067, AS-tDR-009512, and AS-tDR-000076 in HASMC proliferation. GO analysis revealed that the four DEtsRNAs were involved in regulation of complement activation (ontology: biological process, GO: 0030449), actin cytoskeleton organization (ontology: biological process, GO: 0030036), regulation of tumor necrosis factor-mediated signaling pathway (ontology: biological process, GO: 0010803), negative regulation of cell migration (ontology: biological process, GO: 0030336), and negative regulation of smooth muscle cell proliferation (ontology: biological process, GO: 0048662) (Additional file 8). In addition, pathway analysis revealed that the four DEtsRNAs were enriched in Notch signaling pathway (hsa04330), adenosine monophosphate-activated protein kinase (AMPK) signaling pathway (hsa04152), vascular endothelial growth factor (VEGF) signaling pathway (hsa04370), RNA polymerase (hsa03020), and arachidonic acid metabolism (hsa00590) (Additional file 8).

### AS-tDR-000067 suppression inhibits HASMC proliferation by elevating p53 levels

**Fig. 4:**
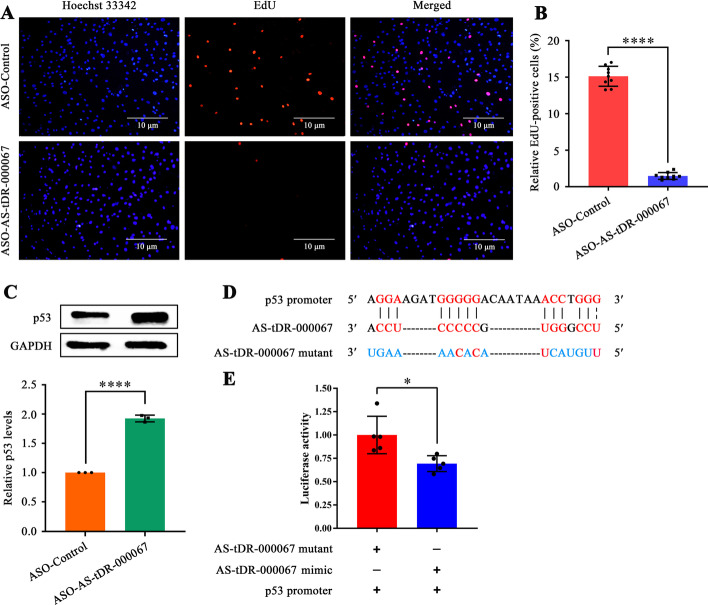
AS-tDR-000067 suppression inhibits HASMC proliferation by elevating p53 levels. **A** EdU incorporation assay of HASMCs transfected with ASO-Control or ASO-AS-tDR-000067. Blue fluorescence (Hoechst 33342) indicates cell nuclei, while red fluorescence (EdU) represents HASMCs with DNA synthesis. Scale bar 10 µm; **B** Relative EdU-positive HASMCs. Data expressed as the ratio of EdU-positive HASMCs to total ones. Data shown as mean ± SD of at least three independent experiments, ****p < 0.0001 versus ASO-Control group; **C** Western blot analysis of p53 in HASMCs transfected with ASO-Control or ASO-AS-tDR-000067, with GAPDH as control. Data shown as mean ± SD of three independent experiments, *p < 0.05 versus ASO-Control group; **D** Schematic of binding sites of AS-tDR-000067 to p53 promoter; **E** Verification of p53 promoter as a target of AS-tDR-000067 utilizing the dual-luciferase reporter gene assay. Data shown as mean ± SD of at least three independent experiments, *p < 0.05

According to RNA-seq and qRT-PCR, AS-tDR-000067 was increased in proliferative HASMCs (Fig. 1D). Antisense oligonucleotide (ASO) specifically suppressed approximately 60% of AS-tDR-000067 expression in HASMCs (Additional file 9). AS-tDR-000067 suppression inhibited HASMC proliferation by 80%, as confirmed by the EdU incorporation assay (Fig. 4A, B). Interestingly, a crucial anti-oncogene, p53 [21], stood out in the AS-tDR-000067-promoter interaction network (Fig. 2B). AS-tDR-000067 inhibition enhanced p53 protein levels in HASMCs (Fig. 4C). Through RNAhybrid [12], we found a potential binding site between AS-tDR-000067 and p53 promoter (Fig. 4D). Dual-luciferase reporter gene assay revealed that AS-tDR-000067 mimic inhibited the fluorescence activity of p53-WT plasmid (Fig. 4E), indicating that the promoter of p53 was a target of AS-tDR-000067. These findings suggest that AS-tDR-000067 promotes HASMC proliferation, at least in part, by inhibiting p53 transcription in a promoter-targeted manner.

### AS-tDR-000076 silencing restrains HASMC proliferation by augmenting MFN2 levels

**Fig. 5:**
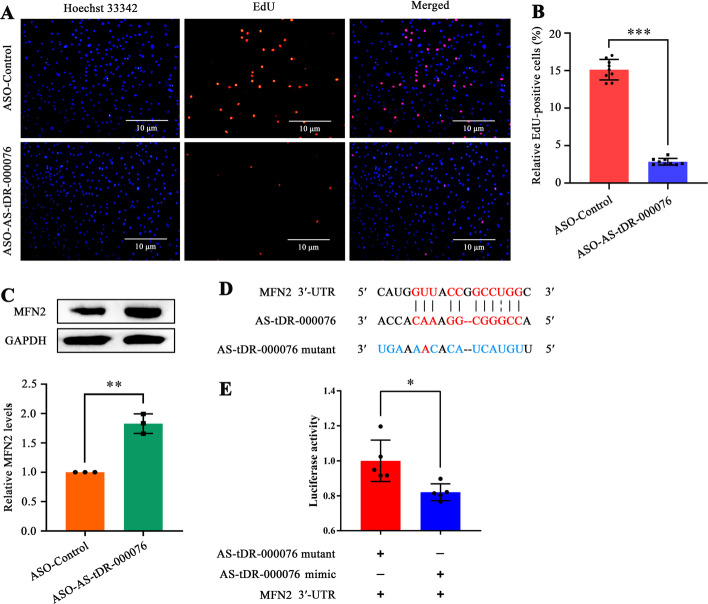
AS-tDR-000076 silencing restrains HASMC proliferation by augmenting MFN2 levels. **A** EdU incorporation assay of HASMCs transfected with ASO-Control or ASO-AS-tDR-000076. Blue fluorescence (Hoechst 33342) indicates cell nuclei, while red fluorescence (EdU) represents HASMCs with DNA synthesis. Scale bar 10 µm; **B** Relative EdU-positive HASMCs. Data shown as the ratio of EdU-positive HASMCs to total ones. Data expressed as mean ± SD of at least three independent experiments, ***p < 0.001 versus ASO-Control group; **C** Western blot analysis of MFN2 in HASMCs transfected with ASO-Control or ASO-AS-tDR-000076, with GAPDH as control. Data shown as mean ± SD of three independent experiments, **p < 0.01 versus ASO-Control group; **D** Schematic of binding sites of AS-tDR-000076 to MFN2 3′-UTR; **E** Confirmation of MFN2 3′-UTR as a target of AS-tDR-000076 employing the dual-luciferase reporter gene assay. Data shown as mean ± SD of at least three independent experiments, *p < 0.05

In the light of the RNA-seq and qRT-PCR results, AS-tDR-000076 was increased in proliferative HASMCs (Fig. 1D). Moreover, GO analysis showed that target DEmRNAs of AS-tDR-000076 were enriched in negative regulation of smooth muscle cell proliferation (ontology: biological process, GO: 0,048,662) (Additional file 8). These two clues suggest that AS-tDR-000076 might be a regulator of HASMC proliferation. ASO specifically restrained approximately 60% of AS-tDR-000076 expression in HASMCs (Additional file 9). AS-tDR-000076 silencing resulted in a reduction of up to 80% in EdU-positive cells (Fig. 5A, B). Intriguingly, MFN2, a crucial VSMC proliferation inhibitor confirmed by our group [22] and others [23–26], is a target gene of AS-tDR-000076 (Fig. 3D). AS-tDR-000076 silencing promoted MFN2 protein levels in HASMCs (Fig. 5C). Utilizing RNAhybrid [12], we found a possible binding site between AS-tDR-000076 and MFN2 3′-UTR (Fig. 5D). Dual-luciferase reporter gene assay revealed that AS-tDR-000076 mimic decreased the fluorescence activity of MFN2-WT plasmid (Fig. 5E), manifesting that the 3′-UTR of MFN2 was a target of AS-tDR-000076. These findings elucidate that AS-tDR-000076 promotes HASMC proliferation, at least in part, by restricting MFN2 levels in a 3′-UTR-targeted manner.

## Discussion

tsRNAs, a new type of noncoding RNAs derived from tRNAs, are receiving increasing attention. tsRNAs are abundant, evolutionarily conserved, and widely distributed, suggesting that they are not the by-products of tRNA production or degradation, and may be involved in organism regulation [27–29]. There are two hot spots in the mechanism of tsRNAs; one is the interaction with proteins [30, 31], and the other is the miRNA-like function that inhibits target gene expression [32]. Through these mechanisms, tsRNAs regulate cell proliferation [33], migration [34], apoptosis [35], and other biological processes [36]. At present, studies on tsRNAs are mainly focused on revealing their roles in cancer [37].

Intriguingly, the mystery that tsRNAs are involved in cardiovascular disease by regulating VSMC proliferation has been gradually resolved [8–10]. tRF^GlnCTG^, which is highly increased in injured rat common carotid arteries, promotes rat VSMC proliferation by inhibiting Fas cell surface death receptor (FAS) levels in a 3′-UTR-targeted manner [8]. tRF-Gly-GCC is increased in patients with atherosclerosis and promotes VSMC proliferation [9]. 5′-tiRNA-Cys-GCA, which is decreased in human and mouse aortic dissection, inhibits VSMC proliferation by reducing STAT4 levels in a 3′-UTR-targeted manner [10]. However, understanding the regulation of VSMC proliferation by tsRNAs is still in its infancy.

Here, we identified 1838 DEtsRNAs between proliferative and quiescent HASMCs using RNA-seq (Fig. 1A–C). Four DEtsRNAs (AS-tDR-001370, AS-tDR-000067, AS-tDR-009512, and AS-tDR-000076) were increased in proliferative HASMCs (Fig. 1D) and mainly located in the nucleus of HASMCs (Fig. 1E). It is worth noting that the regulatory role of tsRNAs in the nucleus has not been well explored, including in VSMC proliferation. Given that miRNAs activate or restrain the transcriptional process in the nucleus by targeting promoters [18, 19], we speculated that tsRNAs might have the same function. We predicted target promoters of the four DEtsRNAs and constructed tsRNA–promoter interaction networks (Fig. 2). Four gene sets containing target promoters were enriched in some VSMC proliferation-related GO terms and pathways (Additional file 7). Analysis of tsRNA–promoter interaction networks indicated that AS-tDR-001370 might target the promoters of cyclin D1 (CCND1) and sprouty RTK signaling antagonist 2 (SPRY2) (Fig. 2A). CCND1 has been confirmed to promote VSMC proliferation [38]. SPRY2 reduces neointimal growth after vascular injury by inhibiting VSMC proliferation and migration [39]. Therefore, AS-tDR-001370 might promote HASMC proliferation by activating CCND1 transcription but inhibiting SPRY2 transcription. Reticulon 4 (RTN4), which inhibits VSMC proliferation and migration [40], contained the target promoter of AS-tDR-009512 (Fig. 2C). Hence, AS-tDR-009512 might facilitate HASMC proliferation by suppressing RTN4 transcription. AS-tDR-000076 was likely to target the promoters of CCND1 and transforming growth factor beta 1 (TGFB1) (Fig. 2D). TGFB1 inhibits VSMC proliferation and promotes VSMC apoptosis [41]. Accordingly, AS-tDR-000076 might accelerate HASMC proliferation by motivating CCND1 transcription but restricting TGFB1 transcription.

Excitingly, we found that the promoter of p53 might be targeted by AS-tDR-000067 (Fig. 2B). p53 is a crucial tumor suppressor gene, and the transcription factor it encodes is essential for the cell cycle and apoptosis [21]. A large number of studies have confirmed that p53 prevents atherosclerosis [42], hypertension [43], vascular stenosis [44], and other vascular remodeling diseases by inhibiting VSMC proliferation, invasion, and migration and inducing VSMC apoptosis. In this study, we revealed that AS-tDR-000067 suppression inhibited HASMC proliferation (Fig. 4A, B) accompanied by augmented p53 levels (Fig. 4C), and further elucidated that the promoter of p53 is a target of AS-tDR-000067 (Fig. 4D, E). Taken together, we document that AS-tDR-000067 promotes HASMC proliferation, at least in part, via inhibiting transcription of p53 in a promoter-targeted manner. However, the mechanism of AS-tDR-000067 translocation into the nucleus is not explored in this work and requires further verification.

In addition, four target DEmRNA sets were also enriched in some VSMC proliferation-related GO terms and pathways (Additional file 8). Investigation of tsRNA–mRNA interaction networks revealed that AS-tDR-009512 might target tropomyosin 1 (TPM1) and transmembrane protein with EGF-like and two follistatin-like domains 2 (TMEFF2) (Fig. 3C). miR-21 promotes VSMC proliferation and migration by targeting and reducing TPM1 [45, 46]. TMEFF2 blocks proliferation of pancreatic cancer cells by inhibiting phosphorylation of the MAPK signaling pathway [47], and the MAPK signaling pathway is highly correlated with VSMC proliferation [48], which hints that TMEFF2 might inhibit VSMC proliferation via the MAPK signaling pathway. Hence, AS-tDR-009512 was likely to promote HASMC proliferation by targeting and suppressing TPM1 and TMEFF2. AS-tDR-000076 was likelihood to target TMEFF2 and splicing factor 1 (SF1) (Fig. 3D). SF1 has been confirmed to inhibit VSMC proliferation [49]. Therefore, AS-tDR-000076 might accelerate HASMC proliferation by silencing SF1 and TMEFF2.

In particular, MFN2 was also a potential target gene of AS-tDR-000076 (Fig. 3D). MFN2 has been verified to restrain VSMC proliferation by our group [22] and others [23–26]. In this study, we uncovered that AS-tDR-000076 silencing restrained HASMC proliferation (Fig. 5A, B) combined with increased MFN2 levels (Fig. 5C), and further clarified that MFN2 is a target of AS-tDR-000076 (Fig. 5D, E). Our data confirm that AS-tDR-000076 accelerates HASMC proliferation, at least in part, via suppressing MFN2 levels in a 3′-UTR-targeted manner.

In our sequencing data, AS-tDR-013295 and AS-tDR-001583 were outstandingly decreased in proliferative compared with quiescent HASMCs. To explore the biological functions of AS-tDR-013295 and AS-tDR-001583, we predicted their target DEmRNAs and constructed tsRNA–mRNA interaction networks (Additional file 10). From the networks, we found that AS-tDR-013295 might target myristoylated alanine-rich protein kinase C substrate (MARCKS) (Additional file 10). MARCKS silencing attenuates VSMC migration and proliferation by increasing cyclin-dependent kinase inhibitor p27 [50]. In addition, AS-tDR-001583 might bind to the myeloid-associated differentiation marker (MYADM) (Additional file 10). MYADM-triggered VSMC proliferation plays a vital role in the development of pulmonary arterial hypertension vascular remodeling via a Krüppel-like factor 4 (KLF4) nuclear export-dependent mechanism, leading to decreased cyclin-dependent kinase inhibitor 1A (p21) levels [51]. Therefore, we speculated that AS-tDR-013295 and AS-tDR-001583 might inhibit VSMC proliferation by suppressing the levels of VSMC proliferation promoters MARCKS and MYADM, respectively. However, this hypothesis needs to be investigated in the future.

## Conclusions

We reveal for the first time a significant difference in tsRNA expression profiles between proliferative and quiescent HASMCs. Furthermore, our results confirm that AS-tDR-000067 promotes HASMC proliferation, at least in part, via suppressing p53 transcription in a promoter-targeted manner. And AS-tDR-000076 accelerates HASMC proliferation, to some extent, via attenuating MFN2 levels in a 3′-UTR-targeted manner. Our results provide clues regarding the regulation of VSMC proliferation by tsRNAs and highlight AS-tDR-000067 and AS-tDR-000076 as significant factors enhancing VSMC proliferation.

## Supplementary Information

**Additional file 1: Table S1.** Primers for qRT-PCR.

**Additional file 2: Table S2.** The gene list containing the target promoters of tsRNAs.

**Additional file 3: Table S3.** The target DEmRNAs list of tsRNAs.

**Additional file 4: Table S4.** Sequences of ASOs.

**Additional file 5: Table S5.** Sequences of tsRNA mimics and tsRNA-mutant.

**Additional file 6: Figure S1.** Successful induction of proliferative and quiescent HASMCs. **A**, **B** Western blot analysis of α-SMA and PCNA between proliferative and quiescent HASMCs, with β-actin as control. Data shown as mean ± SD of four independent experiments, *p < 0.05 versus quiescent HASMCs. **C** EdU incorporation assay of differences in DNA synthesis between proliferative and quiescent HASMCs. Blue fluorescence (Hoechst 33342) indicates cell nuclei, while red fluorescence (EdU) represents HASMCs with DNA synthesis. Scale bar 10 µm. **D** Relative EdU-positive HASMCs. Data expressed as ratio of EdU-positive HASMCs to total ones. Data shown as mean ± SD of at least three independent experiments, ****p < 0.0001 versus quiescent HASMCs.

**Additional file 7: Figure S2.** Functional annotation of genes containing target promoters. GO terms of genes containing target promoters of **A** AS-tDR-001370, **C** AS-tDR-000067, **E** AS-tDR-009512, and (**G**) AS-tDR-000076. Pathways of genes containing target promoters of **B** AS-tDR-001370, **D** AS-tDR-000067, **F** AS-tDR-009512, and **H** AS-tDR-000076.

**Additional file 8: Figure S3.** Functional annotation of target DEmRNAs. GO terms of target DEmRNAs of **A** AS-tDR-001370, **C** AS-tDR-000067, **E** AS-tDR-009512, and **G** AS-tDR-000076. Pathways of target DEmRNAs of **B** AS-tDR-001370, **D** AS-tDR-000067, **F** AS-tDR-009512, and **H** AS-tDR-000076.

**Additional file 9: Figure S4.** Suppression efficiency of AS-tDR-000067 and AS-tDR-000076. **A** qRT-PCR analysis of AS-tDR-000067 expression in HASMCs transfected with ASO-Control or ASO-AS-tDR-000067, normalized by U6. Data shown as mean ± SD of three independent experiments, ****p < 0.0001 versus ASO-Control group. **B** qRT-PCR analysis of AS-tDR-000076 expression in HASMCs transfected with ASO-Control or ASO-AS-tDR-000076, normalized by U6. Data shown as mean ± SD of three independent experiments, ****p < 0.0001 versus ASO-Control group.

**Additional file 10: Figure S5.** Construction of tsRNA–mRNA interaction networks. Subnetworks of **A** AS-tDR-013295 and **B** AS-tDR-001583. Red nodes indicate increased target DEmRNAs, while green nodes indicate decreased DEtsRNAs.

**Additional file 11: Figure S6.** Original images for blots in Fig. 4.

**Additional file 12: Figure S7.** Original images for blots in Fig. 5.

**Additional file 13: Figure S8.** Original images for blots in Fig. S1A.

## Data Availability

The datasets generated during the current study are available in the [GEO] repository at [https://www.ncbi.nlm.nih.gov/geo/query/acc.cgi?acc=GSE164540].

## Abbreviations

AGO: Argonaute
AMPK: Adenosine monophosphate-activated protein kinase
ASOs: Antisense oligonucleotides
CCND1: Cyclin D1
DEmRNAs: Differentially expressed mRNAs
DEtsRNAs: Differentially expressed tsRNAs
EdU: 5-Ethynyl-2′-deoxyuridine
FAS: Fas cell surface death receptor
GAPDH: Glyceraldehyde-3-phosphate dehydrogenase
GO: Gene Ontology
HASMC: Human aortic smooth muscle cell
KEGG: Kyoto Encyclopedia of Genes and Genomes
MARCKS: Myristoylated alanine-rich protein kinase C substrate
MAPK: Mitogen-activated protein kinase
MFN2: Mitofusin 2
MYADM: Myeloid-associated differentiation marker
PCNA: Proliferating cell nuclear antigen
PMSF: Phenylmethylsulfonyl fluoride
qRT-PCR: Quantitative real-time polymerase chain reaction
RTN4: Reticulon 4
RIPA: Radioimmunoprecipitation assay
SF1: Splicing factor 1
SDS-PAGE: Sodium dodecyl sulfate-polyacrylamide gel electrophoresis
smRNAs: Small RNAs
STAT4: Signal transducer and activator of transcription 4
SPRY2: Sprouty RTK signaling antagonist 2
tsRNAs: tRNA-derived small RNAs
TPM1: Tropomyosin 1
TMEFF2: Transmembrane protein with EGF-like and two follistatin-like domains 2
TGFB1: Transforming growth factor beta 1
UTR: Untranslated region
VEGF: Vascular endothelial growth factor
VSMCs: Vascular smooth muscle cells
α-SMA: α-smooth muscle actin

## References

[1] Collaborators GBDCoD (2017). Global, regional, and national age-sex specific mortality for 264 causes of death, 1980–2016: a systematic analysis for the Global Burden of Disease Study 2016. Lancet.

[2] Owens GK, Kumar MS, Wamhoff BR (2004). Molecular regulation of vascular smooth muscle cell differentiation in development and disease. Physiol Rev.

[3] Sun B, Cao Q, Meng M, Wang X (2020). MicroRNA-186-5p serves as a diagnostic biomarker in atherosclerosis and regulates vascular smooth muscle cell proliferation and migration. Cell Mol Biol Lett.

[4] Han Y, Liu Y, Yang C, Gao C, Guo X, Cheng J (2020). LncRNA CASC2 inhibits hypoxia-induced pulmonary artery smooth muscle cell proliferation and migration by regulating the miR-222/ING5 axis. Cell Mol Biol Lett.

[5] Jeong K, Kim JH, Murphy JM, Park H, Kim SJ, Rodriguez YAR, Kong H, Choi C, Guan JL, Taylor JM, Lincoln TM, Gerthoffer WT, Kim JS, Ahn EE, Schlaepfer DD, Lim SS (2019). Nuclear focal adhesion kinase controls vascular smooth muscle cell proliferation and neointimal hyperplasia through GATA4-mediated cyclin D1 transcription. Circ Res.

[6] Matsuoka T, Wada J, Hashimoto I, Zhang Y, Eguchi J, Ogawa N, Shikata K, Kanwar YS, Makino H (2005). Gene delivery of Tim44 reduces mitochondrial superoxide production and ameliorates neointimal proliferation of injured carotid artery in diabetic rats. Diabetes.

[7] Lee YS, Shibata Y, Malhotra A, Dutta A (2009). A novel class of small RNAs: tRNA-derived RNA fragments (tRFs). Genes Dev.

[8] Zhu XL, Li T, Cao Y, Yao QP, Liu X, Li Y, Guan YY, Deng JJ, Jiang R, Jiang J (2021). tRNA-derived fragments tRF(GlnCTG) induced by arterial injury promote vascular smooth muscle cell proliferation. Mol Ther Nucleic Acids.

[9] He X, Yang Y, Wang Q, Wang J, Li S, Li C, Zong T, Li X, Zhang Y, Zou Y, Yu T (2021). Expression profiles and potential roles of transfer RNA-derived small RNAs in atherosclerosis. J Cell Mol Med.

[10] Zong T, Yang Y, Lin X, Jiang S, Zhao H, Liu M, Meng Y, Li Y, Zhao L, Tang G, Gong K, Wang Z, Yu T (2021). 5'-tiRNA-Cys-GCA regulates VSMC proliferation and phenotypic transition by targeting STAT4 in aortic dissection. Mol Ther Nucleic Acids.

[11] Kumar P, Mudunuri SB, Anaya J, Dutta A (2015). tRFdb: a database for transfer RNA fragments. Nucleic Acids Res.

[12] Kruger J, Rehmsmeier M (2006). RNAhybrid: microRNA target prediction easy, fast and flexible. Nucleic Acids Res.

[13] John B, Enright AJ, Aravin A, Tuschl T, Sander C, Marks DS (2004). Human MicroRNA targets. PLoS Biol.

[14] Lin JJ, Chen W, Gong M, Xu X, Du MY, Wang SF, Yang LY, Wang Y, Liu KX, Kong P, Li B, Liu K, Li YM, Dong LH, Sun SG (2021). Expression and functional analysis of lncRNAs involved in platelet-derived growth factor-BB-induced proliferation of human aortic smooth muscle cells. Front Cardiovasc Med.

[15] Agarwal V, Bell GW, Nam JW, Bartel DP (2015). Predicting effective microRNA target sites in mammalian mRNAs. Elife.

[16] da Huang W, Sherman BT, Lempicki RA (2009). Systematic and integrative analysis of large gene lists using DAVID bioinformatics resources. Nat Protoc.

[17] Kumar P, Anaya J, Mudunuri SB, Dutta A (2014). Meta-analysis of tRNA derived RNA fragments reveals that they are evolutionarily conserved and associate with AGO proteins to recognize specific RNA targets. BMC Biol.

[18] Li H, Fan J, Zhao Y, Zhang X, Dai B, Zhan J, Yin Z, Nie X, Fu XD, Chen C, Wang DW (2019). Nuclear miR-320 mediates diabetes-induced cardiac dysfunction by activating transcription of fatty acid metabolic genes to cause lipotoxicity in the heart. Circ Res.

[19] Di Mauro V, Crasto S, Colombo FS, Di Pasquale E, Catalucci D (2019). Wnt signalling mediates miR-133a nuclear re-localization for the transcriptional control of Dnmt3b in cardiac cells. Sci Rep.

[20] Maute RL, Schneider C, Sumazin P, Holmes A, Califano A, Basso K, Dalla-Favera R (2013). tRNA-derived microRNA modulates proliferation and the DNA damage response and is down-regulated in B cell lymphoma. Proc Natl Acad Sci U S A.

[21] Muller PA, Vousden KH (2014). Mutant p53 in cancer: new functions and therapeutic opportunities. Cancer Cell.

[22] Zhang R, Han M, Zheng B, Li YJ, Shu YN, Wen JK (2010). Kruppel-like factor 4 interacts with p300 to activate mitofusin 2 gene expression induced by all-trans retinoic acid in VSMCs. Acta Pharmacol Sin.

[23] Chen KH, Guo X, Ma D, Guo Y, Li Q, Yang D, Li P, Qiu X, Wen S, Xiao RP, Tang J (2004). Dysregulation of HSG triggers vascular proliferative disorders. Nat Cell Biol.

[24] Lu Z, Li S, Zhao S, Fa X (2016). Upregulated miR-17 regulates hypoxia-mediated human pulmonary artery smooth muscle cell proliferation and apoptosis by targeting mitofusin 2. Med Sci Monit.

[25] Feng S, Gao L, Zhang D, Tian X, Kong L, Shi H, Wu L, Huang Z, Du B, Liang C, Zhang Y, Yao R (2019). MiR-93 regulates vascular smooth muscle cell proliferation, and neointimal formation through targeting Mfn2. Int J Biol Sci.

[26] Xu L, Hao H, Hao Y, Wei G, Li G, Ma P, Xu L, Ding N, Ma S, Chen AF, Jiang Y (2019). Aberrant MFN2 transcription facilitates homocysteine-induced VSMCs proliferation via the increased binding of c-Myc to DNMT1 in atherosclerosis. J Cell Mol Med.

[27] Li F, Kaczor-Urbanowicz KE, Sun J, Majem B, Lo HC, Kim Y, Koyano K, Rao SL, Kang SY, Kim SM, Kim KM, Kim S, Chia D, Elashoff D, Grogan TR, Xiao X, Wong DTW (2018). Characterization of human salivary extracellular RNA by next-generation sequencing. Clin Chem.

[28] Godoy PM, Bhakta NR, Barczak AJ, Cakmak H, Fisher S, MacKenzie TC, Patel T, Price RW, Smith JF, Woodruff PG, Erle DJ (2018). Large differences in small RNA composition between human biofluids. Cell Rep.

[29] Krishna S, Raghavan S, DasGupta R, Palakodeti D (2021). tRNA-derived fragments (tRFs): establishing their turf in post-transcriptional gene regulation. Cell Mol Life Sci.

[30] Guzzi N, Ciesla M, Ngoc PCT, Lang S, Arora S, Dimitriou M, Pimkova K, Sommarin MNE, Munita R, Lubas M, Lim Y, Okuyama K, Soneji S, Karlsson G, Hansson J, Jonsson G, Lund AH, Sigvardsson M, Hellstrom-Lindberg E, Hsieh AC, Bellodi C (2018). Pseudouridylation of tRNA-derived fragments steers translational control in stem cells. Cell.

[31] Kim HK, Xu J, Chu K, Park H, Jang H, Li P, Valdmanis PN, Zhang QC, Kay MA (2019). A tRNA-derived small rna regulates ribosomal protein S28 protein levels after translation initiation in humans and mice. Cell Rep.

[32] Martinez G, Choudury SG, Slotkin RK (2017). tRNA-derived small RNAs target transposable element transcripts. Nucleic Acids Res.

[33] Huang B, Yang H, Cheng X, Wang D, Fu S, Shen W, Zhang Q, Zhang L, Xue Z, Li Y, Da Y, Yang Q, Li Z, Liu L, Qiao L, Kong Y, Yao Z, Zhao P, Li M, Zhang R (2017). tRF/miR-1280 suppresses stem cell-like cells and metastasis in colorectal cancer. Cancer Res.

[34] Goodarzi H, Liu X, Nguyen HC, Zhang S, Fish L, Tavazoie SF (2015). Endogenous tRNA-derived fragments suppress breast cancer progression via YBX1 displacement. Cell.

[35] Saikia M, Jobava R, Parisien M, Putnam A, Krokowski D, Gao XH, Guan BJ, Yuan Y, Jankowsky E, Feng Z, Hu GF, Pusztai-Carey M, Gorla M, Sepuri NB, Pan T, Hatzoglou M (2014). Angiogenin-cleaved tRNA halves interact with cytochrome c, protecting cells from apoptosis during osmotic stress. Mol Cell Biol.

[36] Krishna S, Yim DG, Lakshmanan V, Tirumalai V, Koh JL, Park JE, Cheong JK, Low JL, Lim MJ, Sze SK, Shivaprasad P, Gulyani A, Raghavan S, Palakodeti D, DasGupta R (2019). Dynamic expression of tRNA-derived small RNAs define cellular states. EMBO Rep.

[37] Zhu L, Ge J, Li T, Shen Y, Guo J (2019). tRNA-derived fragments and tRNA halves: the new players in cancers. Cancer Lett.

[38] Xiao Q, Zhang F, Grassia G, Hu Y, Zhang Z, Xing Q, Yin X, Maddaluno M, Drung B, Schmidt B, Maffia P, Ialenti A, Mayr M, Xu Q, Ye S (2014). Matrix metalloproteinase-8 promotes vascular smooth muscle cell proliferation and neointima formation. Arterioscler Thromb Vasc Biol.

[39] Zhang C, Chaturvedi D, Jaggar L, Magnuson D, Lee JM, Patel TB (2005). Regulation of vascular smooth muscle cell proliferation and migration by human sprouty 2. Arterioscler Thromb Vasc Biol.

[40] Chick HE, Nowrouzi A, Fronza R, McDonald RA, Kane NM, Alba R, Delles C, Sessa WC, Schmidt M, Thrasher AJ, Baker AH (2012). Integrase-deficient lentiviral vectors mediate efficient gene transfer to human vascular smooth muscle cells with minimal genotoxic risk. Hum Gene Ther.

[41] Liu W, Luo M, Zou L, Liu X, Wang R, Tao H, Wu D, Zhang W, Luo Q, Zhao Y (2019). uNK cell-derived TGF-beta1 regulates the long noncoding RNA MEG3 to control vascular smooth muscle cell migration and apoptosis in spiral artery remodeling. J Cell Biochem.

[42] Mercer J, Figg N, Stoneman V, Braganza D, Bennett MR (2005). Endogenous p53 protects vascular smooth muscle cells from apoptosis and reduces atherosclerosis in ApoE knockout mice. Circ Res.

[43] Zehendner CM, Valasarajan C, Werner A, Boeckel JN, Bischoff FC, John D, Weirick T, Glaser SF, Rossbach O, Jae N, Demolli S, Khassafi F, Yuan K, de Jesus Perez VA, Michalik KM, Chen W, Seeger W, Guenther A, Wasnick RM, Uchida S, Zeiher AM, Dimmeler S, Pullamsetti SS (2020). Long noncoding RNA TYKRIL plays a role in pulmonary hypertension via the p53-mediated regulation of PDGFRbeta. Am J Respir Crit Care Med.

[44] Forte A, Finicelli M, Grossi M, Vicchio M, Alessio N, Sante P, De Feo M, Cotrufo M, Berrino L, Rossi F, Galderisi U, Cipollaro M (2010). DNA damage and repair in a model of rat vascular injury. Clin Sci (Lond).

[45] Wang M, Li W, Chang GQ, Ye CS, Ou JS, Li XX, Liu Y, Cheang TY, Huang XL, Wang SM (2011). MicroRNA-21 regulates vascular smooth muscle cell function via targeting tropomyosin 1 in arteriosclerosis obliterans of lower extremities. Arterioscler Thromb Vasc Biol.

[46] Jia S, Ma WD, Zhang CY, Zhang Y, Yao ZH, Quan XH, Guo X, Wang CX (2019). Tanshinone IIA attenuates high glucose induced human VSMC proliferation and migration through miR-21-5p-mediated tropomyosin 1 downregulation. Arch Biochem Biophys.

[47] Han H, Zhan Z, Xu J, Song Z (2019). TMEFF2 inhibits pancreatic cancer cells proliferation, migration, and invasion by suppressing phosphorylation of the MAPK signaling pathway. Onco Targets Ther.

[48] Won KJ, Lee KP, Baek S, Cui L, Kweon MH, Jung SH, Ryu YK, Hong JM, Cho EA, Shin HS, Kim B (2017). Desalted *Salicornia europaea* extract attenuated vascular neointima formation by inhibiting the MAPK pathway-mediated migration and proliferation in vascular smooth muscle cells. Biomed Pharmacother.

[49] Cattaruzza M, Schafer K, Hecker M (2002). Cytokine-induced down-regulation of zfm1/splicing factor-1 promotes smooth muscle cell proliferation. J Biol Chem.

[50] Monahan TS, Andersen ND, Martin MC, Malek JY, Shrikhande GV, Pradhan L, Ferran C, LoGerfo FW (2009). MARCKS silencing differentially affects human vascular smooth muscle and endothelial cell phenotypes to inhibit neointimal hyperplasia in saphenous vein. FASEB J.

[51] Sun L, Lin P, Chen Y, Yu H, Ren S, Wang J, Zhao L, Du G (2020). miR-182-3p/Myadm contribute to pulmonary artery hypertension vascular remodeling via a KLF4/p21-dependent mechanism. Theranostics.

