# Supplementary material for: Integrated analysis of tRNA-derived small RNAs in proliferative human aortic smooth muscle cells
Source: Cell Mol Biol Lett. 2022 Jun 15;27:47. doi: 10.1186/s11658-022-00346-4 (PMC9199163; doi:10.1186/s11658-022-00346-4)
Supplement: Supplementary file 1 — Additional file 1: Table S1. Primers for qRT-PCR. [file 11658_2022_346_MOESM1_ESM.docx]

##### Supplementary Table 1. Primers for qRT-PCR.

| **RNA** | **Primer sequence (5′ to 3′ )** |
| --- | --- |
| AS-tDR-001370 | Forward primer: ACCTCAGTCGGTAGAGCATCAGA |
| AS-tDR-000067 | Forward primer: CGGGTGCCCCCTCCA |
| AS-tDR-009512 | Forward primer: ACCCACGCGGGAGACC |
| AS-tDR-000076 | Forward primer: TTCGGGCGGAAACACCA |
| U6 | Forward primer: CGATACAGAGAAGATTAGCATGGC |
|  | Reverse primer: AACGCTTCACGAATTTGCGT |
| GAPDH | Forward primer: AGCCACATCGCTCAGACAC |
|  | Reverse primer: AACGCTTCACGAATTTGCGT |
