# Supplementary material for: Integrated analysis of tRNA-derived small RNAs in proliferative human aortic smooth muscle cells
Source: Cell Mol Biol Lett. 2022 Jun 15;27:47. doi: 10.1186/s11658-022-00346-4 (PMC9199163; doi:10.1186/s11658-022-00346-4)
Supplement: Supplementary file 2 — Additional file 2: Table S2. The gene list containing the target promoters of tsRNAs. [file 11658_2022_346_MOESM2_ESM.docx]

##### Supplementary Table 2. The gene list containing the target promoters of tsRNAs.

| Symbol | Count | Target gene |
| --- | --- | --- |
| AS-tDR-001370 | 243 | TLK2, WDR36, RNF40, GNAS, DHCR7, ECT2, TBC1D2, MYD88, GRB10, BATF3, GNS, GPSM1, DLG4, USP13, IGF2BP1, ASIC1, LRRC1, NUP50, LASP1, ANKRD17, ZNF598, PPIG, CDCA2, ZC3H14, GANC, UBE2K, ATP2A2, IDI1, RANBP3, PWWP2B, ZMYND8, ABL1, ENPP1, DST, C6orf62, TNFAIP8L1, ZNF263, PHF21A, ZNRF1, BIN1, PLTP, FOXN2, RALBP1, IBTK, CCDC22, EIF4G2, C3, ADCY6, EIF4ENIF1, PRSS3, MADD, FNBP1, ACTN4, PPP2R5E, FGF5, LRRC37A3, ARID4B, TCTN3, RAD23B, HERC3, POLR3E, PFKFB4, RBMS2, UBE2C, DNAL1, PATZ1, EPB41L2, FAF2, EIF4G1, WDR11, ZFP3, MTMR3, P4HA2, MAP3K4, SLTM, PDE12, NACAD, SETD1A, HNRNPUL1, SLC25A25, GLUD2, DUSP4, IL6ST, C2CD2, MKNK2, SMC3, CDK11A, WDR62, MNT, ACOX1, TBX2, PIH1D2, QSOX1, USP9X, VPS26B, ARHGAP39, PLD1, CNR1, SERPINE1, CDH13, KTN1, TMEM59L, EXT1, RNF123, B4GALT6, UHRF1BP1, CD164, PGAP1, SAP130, SV2A, UBA1, CPSF2, HDAC7, CACNA1G, DDB1, SLC22A4, TUBG1, TDRKH, FAM219A, CRY1, SMARCA1, DDX54, OSBPL3, BMPR2, SPOPL, AGFG1, TNPO2, ZNF197, ADRA1B, NUB1, NADK, USP51, GRIK3, HIPK2, EYA3, RAD23A, BHLHE40, MLPH, IL4R, SUV39H2, TRIM25, COX7A1, MAP1S, PHRF1, SPRY2, DPAGT1, IL1R1, RSRC2, CCNI, IRS2, SNRNP70, RBMS3, UBTF, CHMP7, RNF24, CDC42EP3, SCYL1, FSCN1, FGF9, FRYL, PDE6B, RCAN3, RAVER1, CYB5R3, REPS1, KLF10, FUS, HIVEP2, SF3B2, INPP5B, GRAMD1C, ZNF143, ZBTB7C, TPP1, SEC24C, MSL2, ZC3H3, POLR3B, HSPA1B, CUL9, FLRT2, TRIB1, PPM1B, RNF10, ELOVL4, ZNF425, DDHD2, TOE1, GK, MYO6, ZBTB9, OTUD6A, SLC2A1, MARVELD2, PRKCE, EIF3A, FASN, RETSAT, VPS53, CCND1, RELA, CMKLR1, PPL, MORC2, IPO7, RFX3, TBC1D23, MCAM, ACTG1, FIGNL1, BCAR3, SYNPO, DCP1B, CDV3, TYK2, CASC3, PPP5D1, RALGPS1, MAGEH1, EFCAB7, HOXC4, ALOX5AP, CSMD2, CALML4, RCC1, C2orf80, PNPLA5, TOR4A, RPUSD4, TKTL1, ST14, TNFAIP8, CRADD, ZNF446, SEMA6C, SLC29A2, CCND3, PPIE, SPRED3, NAA38, DYRK1A, ELP6, IKZF1 |
| AS-tDR-000067 | **855** | BRAT1, FXR1, OSMR, HNRNPH3, CAPN10, KAT6B, QRICH1, DLC1, LAMA4, TACC1, CDH18, DHCR7, KLF3, TBC1D2, ITGA9, GRB10, CALD1, p53, YTHDF3, OBSL1, SMARCD1, IARS2, PTP4A3, PPP6R3, NFE2L2, SNX13, KIF1B, TET2, BATF3, PLOD1, BCL2L11, WNK1, ACOX3, ATP11C, ZCCHC24, TMEM214, MAPK9, HIVEP1, PAM, PLXND1, TANC1, GPSM1, SHC1, CAST, HMGA2, USP5, GAPVD1, ZNF438, HTR7, HK1, ALS2, MAD1L1, ZFYVE28, TUBB2A, YY1AP1, KLF6, CSF1, DTNB, VPS26A, MBNL1, MIB2, LGALS8, RPGR, SHB, HIPK1, AKAP12, MME, IGF2BP2, FLNA, LRRC1, HMGA1, DGKD, PERP, ECE1, FOSL1, DESI2, ECHDC1, WWC3, IFITM3, CDCP1, SPRY4, CNNM3, SMPD4, RRM2, ACTA1, RRM1, MRPL33, SCMH1, PKNOX2, CDCA2, PARVA, WDTC1, ITPRIP, HOXD9, UBE2K, PEG10, IDI1, PHKA2, SRSF11, CIZ1, FCHSD2, HYOU1, ZNF451, SMG7, ATG4B, ZBTB44, AMPD3, ABCG2, CXCL12, EEF1B2, MICB, CSGALNACT1, PWWP2B, CADPS2, CAPN5, MAFK, PSAP, WAC, BOLA3, GPR137, SNRPG, ABL1, RAC1, EIF4H, ENPP1, RTN3, DST, GANAB, MAP2, REPIN1, FAM214B, PPFIA1, CHST3, SLC44A1, CUX1, C6orf62, ANKIB1, ATP1A1, CLIP1, PPP1R10, RIPK2, LRSAM1, PLOD2, HPS1, TIMP1, ITPR1, JARID2, PHF21A, SASH1, SLC22A5, IGFBP1, KIF24, TBL1X, BIN1, NRP2, GTF2I, APLP2, ABCB6, GAB3, PRKAR2A, SESN2, MAP3K13, LOXL2, PARD3, HNRNPD, TRIM52, DNM1L, NRG1, IBTK, ANTXR1, TNS3, CCDC22, CEP104, ZBTB24, CTTN, EIF4G2, MANSC1, BCL11A, PPP2R1B, ADCY6, ELN, ZYG11B, TP53BP2, TUBA3D, PRSS3, CAMSAP2, NNT, ANKRD53, MADD, GPIHBP1, TXNRD1, ITPRIPL1, MTAP, FNBP1, RAB3GAP2, ZNF445, TRAPPC11, TMEM181, NRBP2, ERP44, MAPKAPK2, USP42, FGF5, NOC2L, MYLK4, ARID4B, GSR, RPS18, ERC1, RAB11FIP5, COL11A1, AFF4, STRBP, MSMO1, RAD23B, PDZD8, SREK1, YEATS2, PFKFB4, HMMR, SRM, FAM193A, TM9SF3, YAP1, PAFAH1B2, CNOT2, PDGFRA, NINJ1, IPO13, ERRFI1, C2orf81, DVL3, IRF2BP2, NTSR2, KLHL2, GATAD2B, EPB41L2, RGN, CCDC6, RELN, TMEM129, CCDC88A, WDR11, PTGER4, MRPL22, PCSK7, EIF5B, EHBP1L1, GOLIM4, MAN2B2, IER5L, LDLRAD3, P4HA2, EPAS1, MAP3K4, KIAA0754, FGD6, PITPNM1, SVIL, CREM, HSPA9, SLC25A25, C1RL, S1PR3, EXOC6B, ZNF318, BCOR, FRMD8, PXDN, DUSP4, IL6ST, SEC16B, POU6F1, TCP1, PAICS, MON2, ORC4, HUWE1, JAK1, ZDHHC14, SCD, SMC3, CDK11A, CAMKK2, PRPF40B, LZTS2, RAD17, KCNQ5, BMP1, SH3BGRL3, RASAL2, SLC12A2, ABTB2, ZC3HAV1, RAPGEF2, CKS2, SCUBE3, ANKS1B, AZIN1, CHD1, GAB1, NHSL1, UBA5, QSOX1, EPHA5, SORBS3, AHNAK, SATB1, ARHGAP39, ASAP2, PLD1, MAN1A2, CNR1, GIT2, TBXAS1, COL4A5, ADAM19, CD44, PEX3, CFH, LSAMP, HNRNPUL2, AEBP2, ZNF185, CALCA, PARD3B, TBC1D1, HADHB, UBR2, FADS2, MTO1, ZFP91, EXT1, MAP3K11, CMTM8, HSPG2, DSP, RNFT2, SLC9A6, RPS6KA2, UHRF1BP1, IRAK1, SHPRH, COX5B, CD164, PPP1R18, NPDC1, SPTAN1, FAM117B, PCGF5, ARID1A, ULBP2, TJP2, ACBD3, ARHGEF11, FRA10AC1, FGFR1, TPBG, SAP130, SV2A, KCNC4, FSTL1, GPR107, TGFBRAP1, HDAC7, COL5A1, ZSWIM6, PPP1R3G, TEAD1, ETV6, DDB1, HHIP, SLC9A7, UBR3, SLC22A4, IDUA, TDRKH, GNB1, FAM219A, ALDH18A1, SMARCA1, PANK4, ENTPD4, BNC2, TGFBR3, ATP6V0A4, SOS1, SPTBN1, BMPR2, RBM33, ADD3, SERTAD2, B3GALNT2, TMEM67, MRPS17, PRDM8, RMND5A, PDE4B, CDK16, TAPBP, SLC7A2, SPOPL, TSPYL2, TGFBR1, DAP, ABCF1, KANK1, AGL, SLC35A2, NUB1, H6PD, PRRC2A, KCNK1, NADK, VCL, CLSTN1, KIDINS220, USP51, AARD, GRIK3, MAPRE3, NCOA2, SLC25A43, TSPAN5, LOXL4, PHC2, GSTA3, GALNT2, ABCB10, BHLHE40, EXOC3, ACTG2, RAB6A, CAMK2D, MLPH, MARK2, IGF2R, ATN1, PAPSS2, RFC1, RHOBTB2, TNKS, F8A1, UBQLN1, NCOA1, PARP8, BRPF1, FLNC, DIAPH1, GNB3, SUV39H2, TMEM248, MACF1, PHRF1, SLC6A6, BRCC3, DPYD, FLNB, IL1R1, COL13A1, GPR153, FABP5, PTPRF, PLEKHA5, CCNI, ZSCAN20, SNRNP200, ATAD3A, CHST15, ADD1, LEMD2, RIC8B, SEC61G, ADAMTSL4, RAD54L2, FAT1, TMEM41A, HTT, ENO2, NAV1, CDC42EP3, STAM2, MAN1A1, CRIM1, HDLBP, FGD1, TUBB4B, ASTE1, CASK, SMG5, DHRS9, FSCN1, SRPX, HPCAL4, MPP1, BASP1, GFPT1, PTPN12, CERS6, CDC42EP2, EZR, AKT3, MICAL1, AMBP, MTF1, BZW1, PDE6B, RCAN3, CSNK2B, ASAP3, CITED4, ATP2C1, RHOBTB3, TSPAN14, PTDSS1, GPC1, DLG3, COL3A1, NCS1, TRIM44, IL18R1, RABGAP1L, LPCAT1, PKP3, NCAPD2, FOXK1, LRRC8C, EPHA4, HIP1, WDR47, CLOCK, EDIL3, REPS1, LRRN2, PSIP1, EXTL2, MALL, NCBP1, KLF10, GPR137B, ATP8B2, FYN, FAM199X, HIVEP2, DBN1, ABCC5, NOL8, SPPL3, INPP5B, TTLL7, RAB3GAP1, DCBLD2, ATRIP, NOLC1, YME1L1, YWHAG, KCND1, CAPRIN2, VEGFC, PLCB3, ADAM8, ATP2B1, TPP1, LYAR, MSL2, PICALM, LIMD1, POLR3B, NSD1, HSPA1B, DCHS1, ARHGEF2, LOX, SH3RF1, PABPC1, LRRFIP2, VLDLR, TRIB1, MEN1, CPT1A, ATG4A, ZBTB16, LRRC8A, ELOVL4, ZNF425, HDAC4, C11orf24, TOE1, PTPN13, SH2D4A, TSC1, PNMA2, SERPINB9, TNFRSF10D, FBXO11, FBLN2, MARK1, MYLK, MIA3, NOL6, GNAQ, ZFAND5, TCF7, TBC1D9B, CDK13, PSMD1, SLC38A2, PAPPA, TBX18, KDM6A, SERPINE2, SLC5A9, SYNE1, PTPRB, MDH2, AMOTL1, PRKCE, RETSAT, CDK6, ZDHHC2, IFITM10, ITGA4, APBB2, INTS9, TRIM11, NOP14, ETFDH, HIPK3, SEL1L3, SLC36A4, DOCK5, CLDN19, EHMT2, GNAI2, SIX2, RBBP5, CNOT6, RELA, IGF2, MAP9, HEG1, PYGM, TCIRG1, SFRP1, NCAPD3, TMEM43, ELK3, APBB1, TIPARP, AFAP1L1, PTGS1, CDC25C, UBASH3B, RAD18, ZNF398, PRDM4, MYO10, MCAM, NUDC, KDR, UHRF2, LRRFIP1, NIPBL, ARHGEF17, DHCR24, CIT, GTF2IRD1, SCARB1, SLC30A5, KIF21A, CPEB3, CPT2, VCAN, SF1, RAD21, CDCA8, FAM91A1, KIAA0040, FIGNL1, BCAR3, PDE4DIP, ZNF654, ADAM12, FAM167A, TRRAP, PREP, PEX14, ZFAND3, TEAD3, SYNPO, G6PD, TRAK2, FBXL4, OTUD7B, PHF13, PHF2, WDR26, ACAP2, PARP1, DCP1B, CDV3, EPHB2, KIAA2026, ASPM, ARL4C, MGAT4C, RALGPS1, HTATIP2, PARP9, IL11RA, CD96, HOXC4, DRD5, TAF11, ICA1L, CSMD2, BARD1, HILPDA, CCK, ZNF32, CALM2, PGBD2, MYNN, C4orf47, RCC1, RERG, C8orf48, SGPP2, RBP1, PRSS35, ZNF511, SPI1, ELF5, ATP6V1G2, MLXIPL, UBE2E1, POM121L12, ANXA3, CTSK, DPP6, OPRM1, POU4F2, CELF2, SFTPB, TNNT3, SPON2, TOR4A, PGA4, DLK2, BEX4, TAF9B, SETD9, NGF, BEND5, LCN15, SLC39A14, PTPRO, LHX9, ST14, C1orf21, SRBD1, ACOXL, RGS3, NKX2-5, C1orf210, TNFAIP8, SLC6A9, TATDN3, RAET1G, FOXP4, OLIG3, CAMK2A, FAM71F1, PQBP1, NAMPT, GRHL3, TRIQK, LGALS12, GLIS1, BTC, TMEM42, ART5, FHL3, PIK3R1, LYNX1, WIPI2, KHDC1, CTHRC1, KIF3A, IKBKG, FEV, WWC1, OPN1LW, PPP1R9A, BTBD11, SSX2, PDE5A, PSCA, ENG, HOPX, LMX1B, PHB2, CCND3, CD8B, MFAP2, CD5, FANK1, RASSF1, GNLY, MED7, KLKB1, CDK2, BRF2, NLRP3, GRIA1, ASCL2, BEND2, NIPSNAP3B, CAMLG, MOB1A, MTHFD1L, XRCC4, ATP1A4, DUSP13, VEGFB, RWDD3, DDR1, ELP6, ASB12, PYROXD2, PLA2G2E, HOXD13, SYBU, SPRR4, IRF6, PHYHD1, ZMYM3, FAM110B, SLC2A14, SLC29A4, PIGY, ZNF596, SLC40A1, PLCH1, BTF3, ANKRD37, SLC15A3, IRAK1BP1, EDF1, MED30, IKZF1, SPATA9, SYK, C3orf38, MANEAL |
| AS-tDR-009512 | **202** | CAPN10, DLC1, CALD1, NOD1, PRRC1, ACOX3, TMEM214, TANC1, GPSM1, PSAT1, FICD, NOP9, HK1, MED12, RPGR, SLC30A1, C9orf47, CDCP1, BRD7, LETM1, PPP2R5A, STK36, HOXD9, DPP4, IDI1, ATG4B, AMPD3, WAC, ADNP, SYT1, SP100, DST, GANAB, FAM214B, PPP1R10, KIFC3, MFSD12, JMY, ITPR1, AGGF1, SASH1, PHLDB1, DHX38, CNDP2, SESN2, MAP3K13, CNOT4, RALBP1, EFHC1, ELN, PRSS3, GPT2, TXNRD1, VAMP3, FGF5, CNOT2, KLF13, UBE2C, ARRDC4, HSPA5, CACNB2, CNOT3, BUB1, MAN2B2, MID2, NR2C1, MOG, S1PR3, HSPB6, IL6ST, PRSS1, ORC4, PRKCA, HMSD, LZTS2, PALMD, WWP2, HGC6.3, ASAP2, COL4A5, KDM3B, ZKSCAN2, IL1RL1, HLCS, JAZF1, MRPL32, COX5B, FAM117B, SV2A, OSBP, OSBPL3, RBM33, MCM5, CAPN2, TBX1, SLC35E1, RRBP1, SLC35A2, NADK, RERE, NAV2, ACTG2, RTN4, CEP152, PTPRF, SF3A1, MPP5, AKAP8L, TSC22D1, RNF24, KPNA2, NR2F1, ZNF862, RELB, SMG5, GPR176, ASAP3, GTF3C1, PRKACA, DLG3, LPCAT1, PDK4, TAOK3, TRIM54, FYN, CKAP2, GRAMD1C, PICALM, KIF16B, DDHD2, MTMR12, DIDO1, GYS1, PITPNM3, STK24, SYNE1, DHX15, HIPK3, AMPH, CLSTN3, COL1A1, RAB6C, UBASH3B, CLMP, STK11, PHF12, DGCR2, INF2, SYNPO, XPNPEP3, POGZ, MLLT11, FOS, PARP1, TYK2, MXI1, MCHR2, MGAT4C, ZMAT4, HTATIP2, MPZL3, SYT4, ADORA2A, RIBC1, OMD, ZNF32, KLK15, RPL31, CR1, RBP1, KRTAP6-3, TMEM208, TRIM17, DPP6, CELF2, BDH2, HGD, EEF1E1, FAM71F1, PRR19, CRADD, TCEAL1, LTK, LGALS12, FBLN7, BTC, PCSK6, HDDC2, SLC29A2, IKBKG, PHB2, MFAP2, PTPRH, ANKRD1, ZNF415, N4BP2L1, EIF4A1, WDR89, KRCC1, SLC2A14, COQ10A, BST1 |
| AS-tDR-000076 | **121** | AFF4, MMD, ECHDC1, GOLT1B, DIPK2A, ATG4B, B4GALT6, LRRC18, ERBIN, RBM28, ST3GAL1, CCND2, CROT, DSP, EYA3, CASKIN2, CDC27, TNFAIP1, RAD54L2, MAN2C1, LEMD3, MARCHF1, HNF4A, SCUBE3, YAP1, BICD1, OSBPL3, KLF13, ZYX, TGFB1, HERPUD1, GPC1, IMMT, BPTF, PLEKHH2, PREX1, RPL28, PTPA, RILPL1, SH3BGRL3, MUC1, MAP4K5, PERP, SEC23A, MAPK9, ETFRF1, WASHC5, LAMC2, EIF1AX, RERE, PRKCE, TUT7, C6orf15, BCDIN3D, BCR, C17orf80, SCRG1, COPS2, PLA2G2E, SLC2A14, SVIL, ADNP, CD80, CRYBG3, TAOK2, P3H1, AMT, SETD1A, PRKX, TSR1, FBXW5, SYMPK, PTPRU, SFRP1, TIMP2, GPATCH8, SETMAR, MBNL1, XPNPEP3, CACNA1H, SLC25A1, SEMA6C, CAPN10, ZBTB4, SUPT5H, TMEM17, MAML3, MBD1, SOS1, PYROXD2, GNB1, MIA3, IER5L, JUP, TET2, RHOBTB3, HSFX1, SPOPL, POFUT1, ZNF596, KAT6B, SRP72, DCHS1, EHD2, ZFP36L1, CAMK2D, PTPN11, SLIT3, SHMT1, CCND1, ADD3, VPS35, EIF3CL, DNM1L, S1PR3, MANEAL, CNOT1, PNMA8A, SYNRG, CT45A6, IGF2BP2 |
