# Supplementary material for: Integrated analysis of tRNA-derived small RNAs in proliferative human aortic smooth muscle cells
Source: Cell Mol Biol Lett. 2022 Jun 15;27:47. doi: 10.1186/s11658-022-00346-4 (PMC9199163; doi:10.1186/s11658-022-00346-4)
Supplement: Supplementary file 3 — Additional file 3: Table S3. The target DEmRNAs list of tsRNAs. [file 11658_2022_346_MOESM3_ESM.docx]

##### Supplementary Table 3. The target DEmRNAs list of tsRNAs.

| Symbol | Count | Target gene |
| --- | --- | --- |
| AS-tDR-001370 | **46** | CNGB1, HOXD11, TMEM235, PIRT, DENND1A, TMEM92, CDH24, DEF8, PHB2, C1orf198, ERCC1, OSM, NNMT, SH3BP2, TULP1, MCHR2, C2, C2orf68, RPP25L, HIST1H2BD, SCRT1, TMEM63A, MAP4, DIO3, PTPRE, RAB3C, ULK1, GTPBP3, MAL, AKIP1, KBTBD11, CLMN, NUMBL, ONECUT3, SPRED3, OSCAR, SHISA6, GRAPL, SRSF10, SOX8, PIGS, HNF4A, B4GALT7, RPUSD4, EIF3G, C11orf54 |
| AS-tDR-000067 | **70** | KCNG1, PRKCG, ANKRD13B, CAPS, FOXJ1, GYPC, ATP1A1, WNT3A, ZC3H6, RALGPS1, IER3, IKBKG, TCF21, JRK, SLC25A34, C19orf81, ART4, CD86, ADAMTS17, GPC4, RHOD, CCS, SLC29A4, MRAS, PSMB11, DNAAF2, GTF2B, SCRT1, SFT2D2, PSCA, PYCARD, THEM6, CA13, PLIN4, SPATA17, PLA2G4E, GABRR2, PAX9, NECAP2, CEACAM18, ENAH, SIM1, SAMD8, KBTBD11, ATXN3, ZDHHC19, HK1, LRFN2, PKNOX1, RALGDS, ARMC10, MR1, ONECUT3, FOXP4, MARCH2, DFFA, OSCAR, ZSCAN2, ENSA, PPIL2, EHD3, LGI3, LYNX1, B4GALT7, KIAA0319, MMP25, TCEA3, TPSD1, TIFA |
| AS-tDR-009512 | **184** | HEPH, ING3, ACSM3, TRIQK, PRAMEF13, ARHGEF9, NAT8L, PIWIL3, SEC23IP, MFN2, PCSK6, OSGEP, MAD2L1, SLC4A5, ASTN2, PI15, TYRP1, NPY, TRAIP, MAP6, KRT34, CGRRF1, ACER3, SEPT7, HFM1, CITED2, OGN, TECTB, RTP2, PRKD2, GPR161, BTBD11, AMDHD1, KNG1, VSTM2A, SYBU, CASKIN2, SLC6A12, PTPRJ, NFYB, RNMT, LMCD1, ATP2B3, N4BP2L1, SCN10A, PTCHD1, TTC33, TRIM5, ZNF519, C1QC, HRK, TBX5, THAP10, COQ7, RERG, RASAL1, HSD17B11, GABRR2, CNGB3, MYNN, IDNK, TMEM74, KLRC2, MRPS18A, PRH2, RNF187, CDC42, CINP, WASF3, LRP10, HCN3, HEBP2, ZMYM3, OSTC, PCDH1, GPR183, SUMO2, BEND2, HIST1H2BG, SERPINB1, SOWAHD, SNTN, EHD3, LRRN3, WFIKKN2, CYP2C8, ELF5, POLR2C, ZMAT4, CES4A, IFT20, RPS6KC1, ZNF678, DMP1, APOBR, TRA2B, TMEM98, ZSCAN16, ADI1, DENND1A, GPD1, PIGP, HOXD13, ZNF583, KLRC1, VWA2, ERCC1, FAM3B, COQ5, HPGDS, CHMP3, HTR5A, LRIG1, HSD17B7, TMEFF2, PCSK2, AGA, HOXB1, PSMD9, YAF2, RCHY1, KIRREL2, SH2D7, SEMA6D, LRRC8B, SP3, SYPL1, UPRT, ZMAT2, NXT2, ZC3H12B, PVR, BLOC1S4, LDHD, SYNPO2, KCTD15, C6orf223, GPX7, NMRK1, IKZF1, CREG2, TLX2, TMEM237, MTMR8, IRF6, STAP1, GOLGA8A, MYCT1, TBXAS1, CAMLG, FAM53C, TPM1, STK33, ADSSL1, MX2, RLN2, DLG2, NFASC, SLC2A12, CD80, RNF26, DDIT3, DDN, SIX4, CRBN, TMEM231, NCKIPSD, EIF4E3, SLC46A1, PDE11A, LILRB5, SEPT8, PSMD13, KCNE2, ORC6, ABLIM3, BCL11A, TACO1, LHX9, GHSR, CTC1, MARCH1, GOLGA8B, RXFP2 |
| AS-tDR-000076 | **307** | KCNG1, PRKCG, ZNF446, HEPH, ING3, CAPS, KIAA1210, ACSM3, TRIQK, PRAMEF13, ARHGEF9, ABR, NAT8L, GYPC, PIWIL3, SEC23IP, MFN2, PCSK6, OSGEP, MAD2L1, SLC4A5, TCF21, ASTN2, PI15, TYRP1, HIST1H3E, JRK, DAND5, NPY, TRAIP, MAP6, KRT34, CGRRF1, ADAMTS17, MATN4, ACER3, SEPT7, HFM1, CITED2, SLC29A4, MRAS, OGN, TECTB, KIAA0513, PSMB11, RNF126, RTP2, PRKD2, GPR161, ARHGEF4, HM13, BTBD11, AMDHD1, KNG1, VSTM2A, SYBU, CASKIN2, SLC6A12, ISLR2, PTPRJ, NFYB, RNMT, LMCD1, SLC25A45, SCRT1, ATP2B3, SFT2D2, N4BP2L1, SCN10A, FUT2, PTCHD1, HIST3H2BB, XKR4, TTC33, ARL5A, TRIM5, ZNF519, THEM6, C1QC, PLIN4, CLN8, HRK, TBX5, THAP10, COQ7, RERG, SPATA17, RASAL1, MGAT1, HSD17B11, KIF18B, GABRR2, PAX9, CNGB3, CEACAM18, MYNN, IDNK, TMEM74, ENAH, KLRC2, MRPS18A, PRH2, SIM1, SAMD8, ATXN3, ZDHHC19, RNF187, HK1, CDC42, CINP, ARMC10, WASF3, LRP10, HCN3, MR1, ACHE, HEBP2, SF1, ZMYM3, FOXP4, SGPP2, DFFA, OSCAR, OSTC, PCDH1, GPR183, SUMO2, BEND2, HIST1H2BG, ZSCAN2, SERPINB1, SOWAHD, SNTN, EHD3, BAG2, LRRN3, FAM83F, LYNX1, WFIKKN2, CYP2C8, SLC35A3, ELF5, MMP25, POLR2C, ZMAT4, TTC9C, CES4A, TPSD1, IFT20, RPS6KC1, ZNF678, MPL, DMP1, NKX2-5, HOXD11, ANKRD13B, ACTR10, APOBR, FOXJ1, TRA2B, TMEM98, ZSCAN16, ADI1, DENND1A, GPD1, PIGP, HOXD13, ZNF583, ATP1A1, KLRC1, WNT3A, ZC3H6, RALGPS1, VWA2, IER3, IKBKG, ERCC1, FAM3B, CREBBP, COQ5, PEX26, SLC25A34, HPGDS, C19orf81, CHMP3, RBM3, HTR5A, LRIG1, HSD17B7, TMEFF2, ART4, UCK1, PCSK2, CD86, AGA, GREM2, HOXB1, GPC4, PSMD9, RHOD, CCS, YAF2, RCHY1, SPI1, KIRREL2, MGAT4C, GABRD, DNAAF2, SH2D7, SEMA6D, GTF2B, LRRC8B, SP3, SYPL1, UPRT, RABL2B, TOR4A, ZMAT2, NXT2, KNCN, PSCA, ZC3H12B, PYCARD, PVR, BLOC1S4, CA13, LDHD, SYNPO2, KCTD15, C6orf223, GPX7, NMRK1, IKZF1, CREG2, PLA2G4E, TLX2, TMEM237, NECAP2, MTMR8, IRF6, MPV17L, STAP1, AKIP1, ANKRD46, GOLGA8A, MYCT1, TBXAS1, NSL1, KBTBD11, CAMLG, FAM53C, TPM1, LRFN2, STK33, PKNOX1, RALGDS, ADSSL1, TADA2A, ONECUT3, MX2, AP5Z1, RLN2, MARCH2, DLG2, EBF4, NFASC, PRIM1, CD80, SLC2A12, RNF26, SORBS1, MAG, DDIT3, ENSA, DDN, PPIL2, SIX4, CRBN, TMEM231, FGFR1OP, MED28, NCKIPSD, EIF4E3, SLC46A1, SALL4, PDE11A, LILRB5, LGI3, B4GALT7, SEPT8, KIAA0319, PSMD13, KCNE2, MYO1G, ORC6, TCEA3, ABLIM3, BCL11A, TACO1, LHX9, TIFA, GHSR, CTC1, MARCH1, GOLGA8B, RXFP2 |
| AS-tDR-013295 | **72** | MLL3, STARD13, IGF2BP1, FCHO2, CSGALNACT1, ETNK1, SLC44A1, FAM20B, EIF4G2, ASPH, UBE2H, CDC27, FBXO28, PDGFRA, DVL3, KIAA0494, SENP1, CPEB2, LUZP1, ZFHX3, UBE3C, SLC12A2, MBD2, FMR1, C18orf25, CAP1, BRWD1, PCGF5, ARF4, FSTL1, MARCKS, BCL6, SRGAP3, RBM33, COL12A1, RMND5A, GRIK3, HIPK2, GRSF1, ANKRD52, SLC6A6, MEF2C, MCL1, IRS2, ELAVL1, SOCS4, CDC42EP3, STX16, FRYL, RANBP2, CTNNA1, ABHD3, KPNA3, LPAR1, PPM1B, ATP2B1, MKL2, ZFAND5, AP3B1, GPHN, SLC36A4, RANBP17, VCPIP1, KCTD12, PURB, IFT80, ZNF746, SPRED2, RILPL1, THRB, FMNL2, MXD1 |
| AS-tDR-001583 | **35** | TLK2, EEA1, CLASP1, MBNL1, MYADM, MAFK, EIF4G2, ARID2, LRP8, SEPT2, CNOT2, DLG1, SERBP1, HNRNPA3, MNT, PCBP2, NFAT5, RPGRIP1L, BRWD1, TC2N, BMPR2, ARSB, ZFP36L1, ANKRD13C, TRPS1, MTF1, QTRTD1, TANC2, EBF1, ZC3H12C, APOOL, KIAA1217, KIAA0355, THRB, DGCR2 |
