# Supplementary material for: Integrated analysis of tRNA-derived small RNAs in proliferative human aortic smooth muscle cells
Source: Cell Mol Biol Lett. 2022 Jun 15;27:47. doi: 10.1186/s11658-022-00346-4 (PMC9199163; doi:10.1186/s11658-022-00346-4)
Supplement: Supplementary file 4 — Additional file 4: Table S4. Sequences of ASOs. [file 11658_2022_346_MOESM4_ESM.docx]

##### Supplementary Table 4. Sequences of ASOs.

| **DNA** | **sequence (5′ to 3′ )** |
| --- | --- |
| ASO-Control | GCCCGGATAGCTCAGTCGGTAGAGCATCAGACT |
| ASO-AS-tDR-000067 | TGGTGTTTCCGCCCGGT |
| ASO-AS-tDR-000076 | TGGAGGGGGCACCCGGA |
