# Supplementary material for: Integrated analysis of tRNA-derived small RNAs in proliferative human aortic smooth muscle cells
Source: Cell Mol Biol Lett. 2022 Jun 15;27:47. doi: 10.1186/s11658-022-00346-4 (PMC9199163; doi:10.1186/s11658-022-00346-4)
Supplement: Supplementary file 5 — Additional file 5: Table S5. Sequences of tsRNA mimics and tsRNA-mutant. [file 11658_2022_346_MOESM5_ESM.docx]

##### Supplementary Table 5. Sequences of tsRNA mimics and tsRNA-mutant.

| **RNA** | **sequence (5′ to 3′ )** |
| --- | --- |
| tsRNA-mutant | UUGUACUACACAAAAGU |
| AS-tDR-000067 mimic | UCCGGGUGCCCCCUCCA |
| AS-tDR-000076 mimic | ACCGGGCGGAAACACCA |
