# Supplementary figures and images for: Integrated analysis of tRNA-derived small RNAs in proliferative human aortic smooth muscle cells
Source: Cell Mol Biol Lett. 2022 Jun 15;27:47. doi: 10.1186/s11658-022-00346-4 (PMC9199163; doi:10.1186/s11658-022-00346-4)

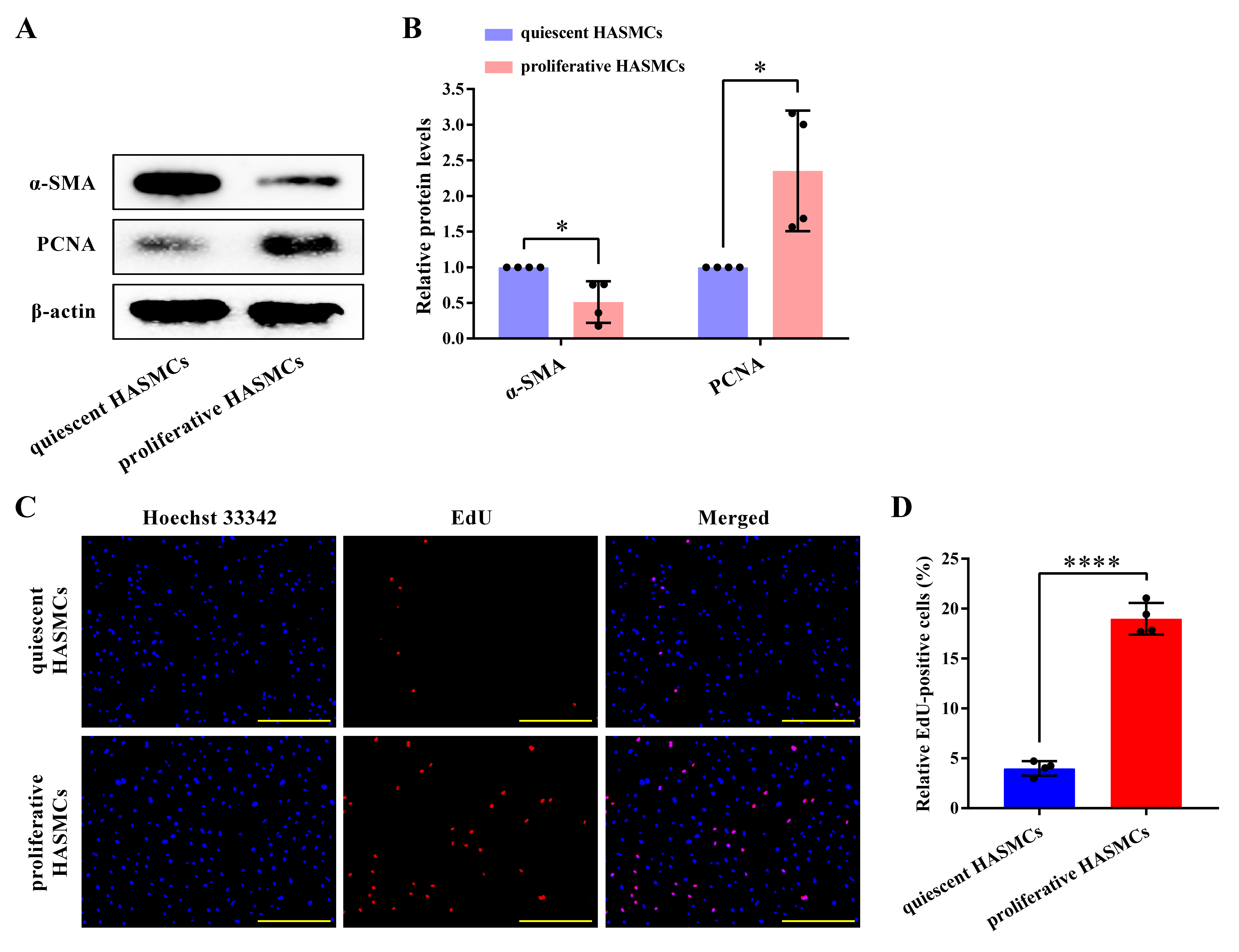

Supplement: Supplementary file 6 — Additional file 6: Figure S1. Successful induction of proliferative and quiescent HASMCs. A, B Western blot analysis of α-SMA and PCNA between proliferative and quiescent HASMCs, with β-actin as control. Data shown as mean ± SD of four independent experiments, *p < 0.05 versus quiescent HASMCs. C EdU incorporation assay of differences in DNA synthesis between proliferative and quiescent HASMCs. Blue fluorescence (Hoechst 33342) indicates cell nuclei, while red fluorescence (EdU) represents HASMCs with DNA synthesis. Scale bar 10 µm. D Relative EdU-positive HASMCs. Data expressed as ratio of EdU-positive HASMCs to total ones. Data shown as mean ± SD of at least three independent experiments, ****p < 0.0001 versus quiescent HASMCs. [file 11658_2022_346_MOESM6_ESM.tif]

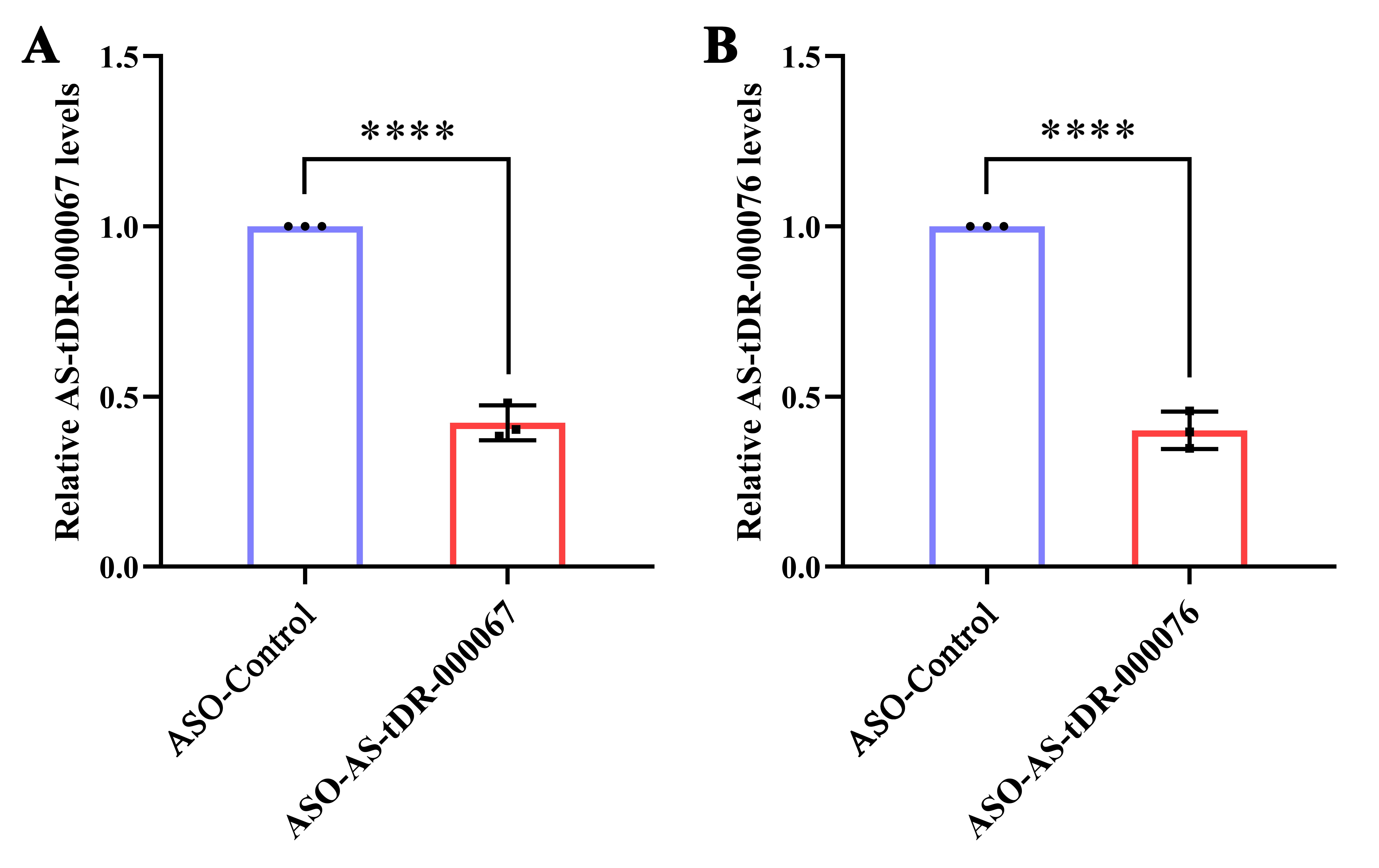

Supplement: Supplementary file 9 — Additional file 9: Figure S4. Suppression efficiency of AS-tDR-000067 and AS-tDR-000076. A qRT-PCR analysis of AS-tDR-000067 expression in HASMCs transfected with ASO-Control or ASO-AS-tDR-000067, normalized by U6. Data shown as mean ± SD of three independent experiments, ****p < 0.0001 versus ASO-Control group. B qRT-PCR analysis of AS-tDR-000076 expression in HASMCs transfected with ASO-Control or ASO-AS-tDR-000076, normalized by U6. Data shown as mean ± SD of three independent experiments, ****p < 0.0001 versus ASO-Control group. [file 11658_2022_346_MOESM9_ESM.tif]

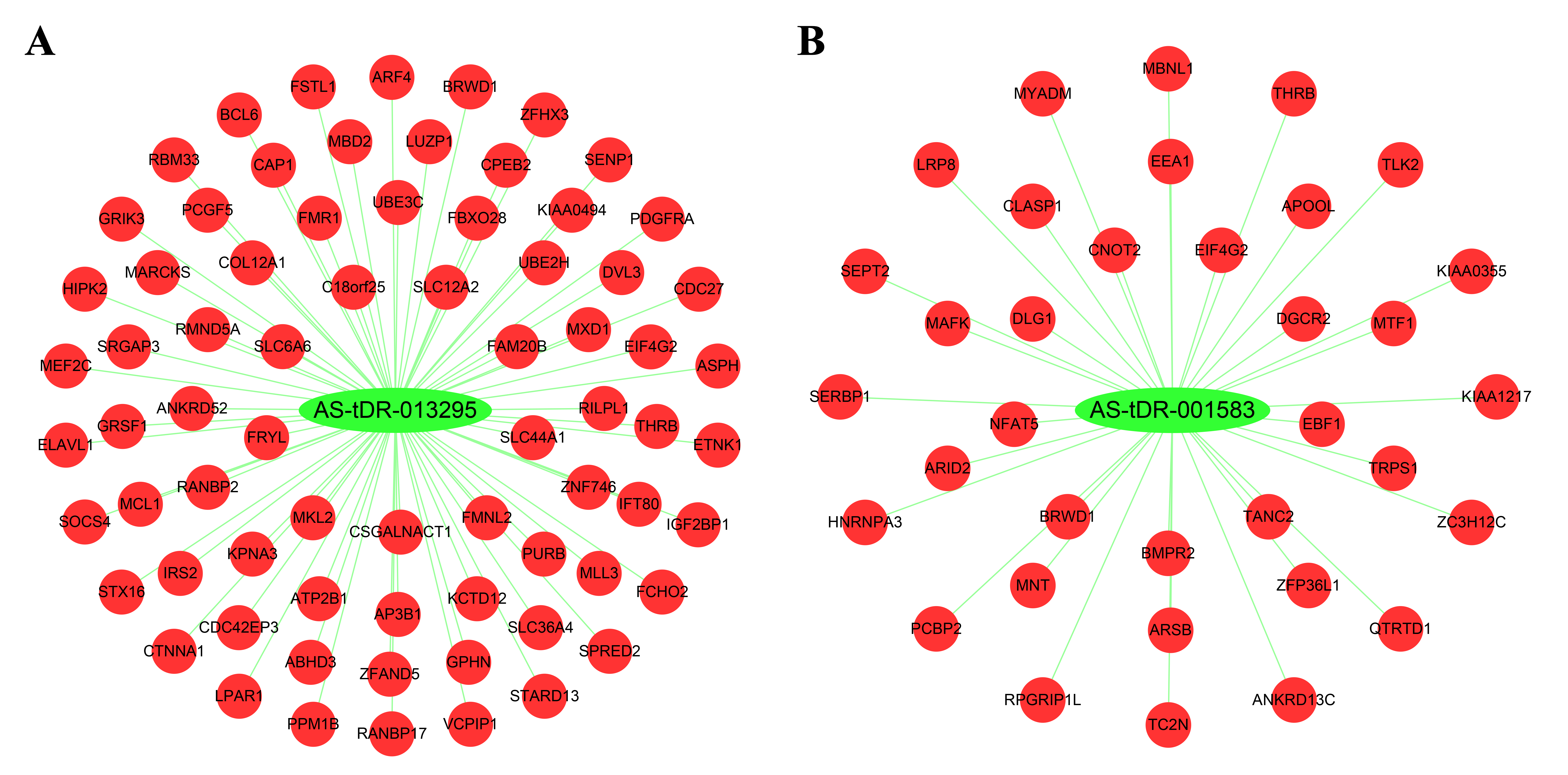

Supplement: Supplementary file 10 — Additional file 10: Figure S5. Construction of tsRNA–mRNA interaction networks. Subnetworks of A AS-tDR-013295 and B AS-tDR-001583. Red nodes indicate increased target DEmRNAs, while green nodes indicate decreased DEtsRNAs. [file 11658_2022_346_MOESM10_ESM.tif]

Original iamges for blots in Figure 4

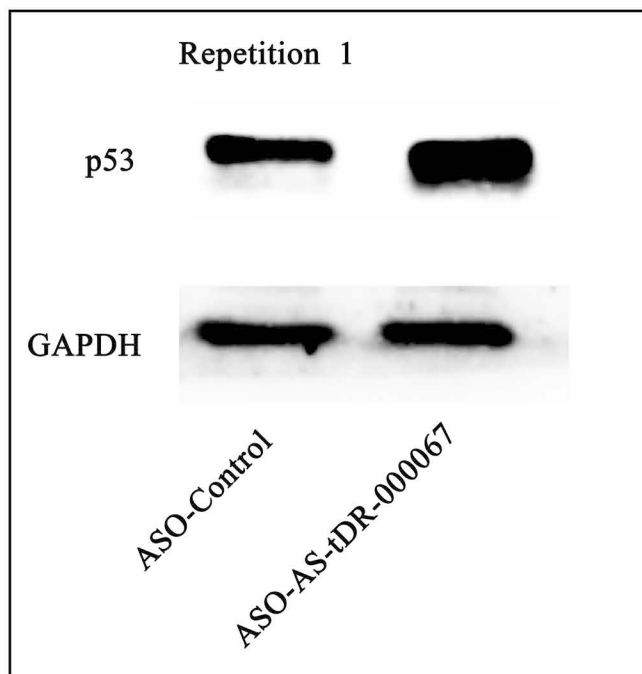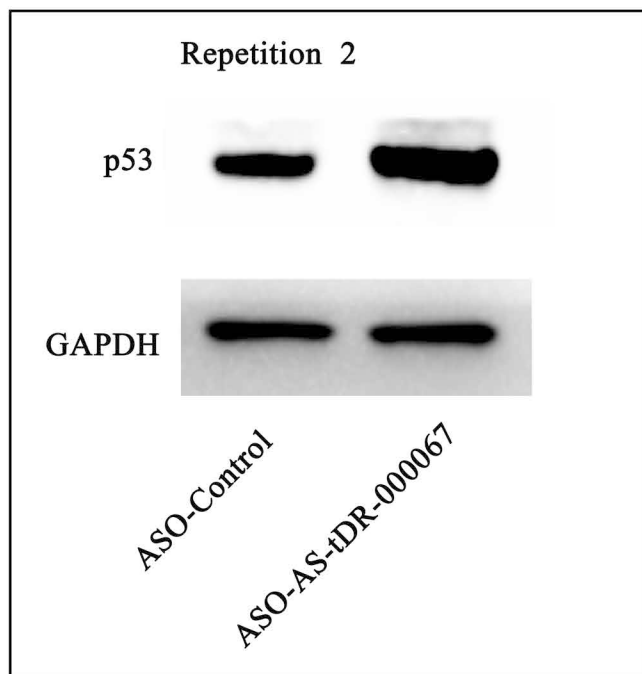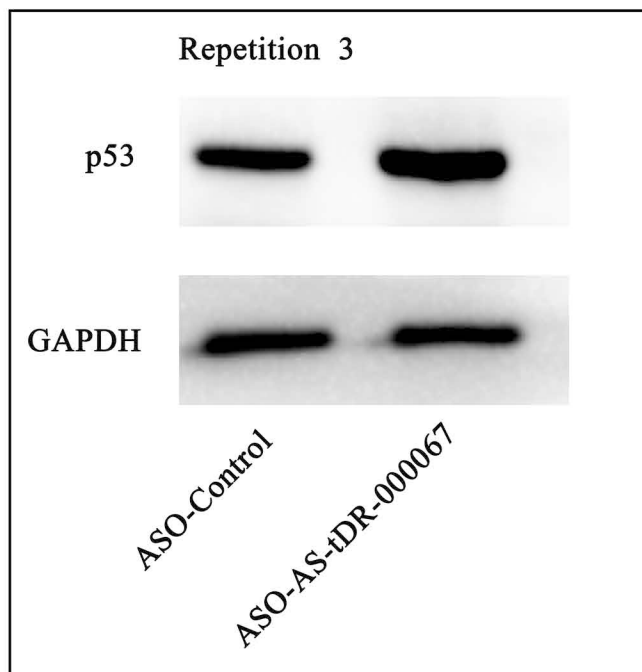

Supplement: Supplementary file 11 — Additional file 11: Figure S6. Original images for blots in Fig. 4. [file 11658_2022_346_MOESM11_ESM.pdf]

# Original iamges for blots in Figure 5

Repetition 1

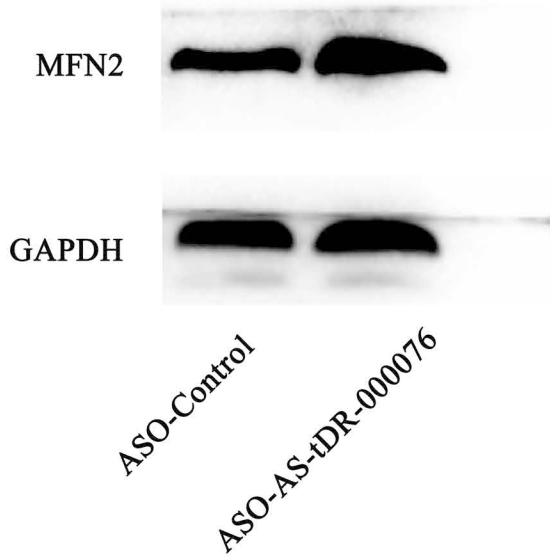

Repetition 2

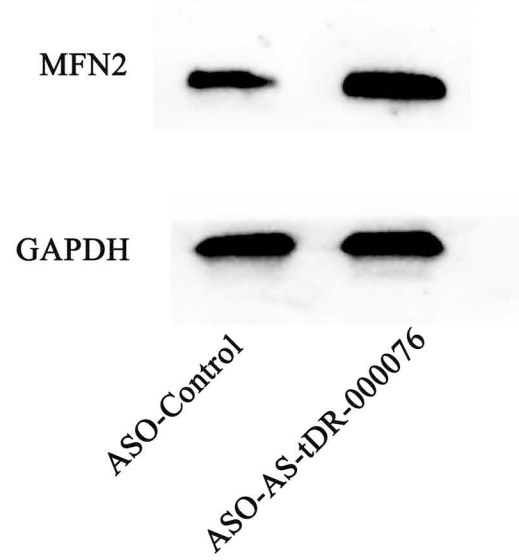

Repetition 3

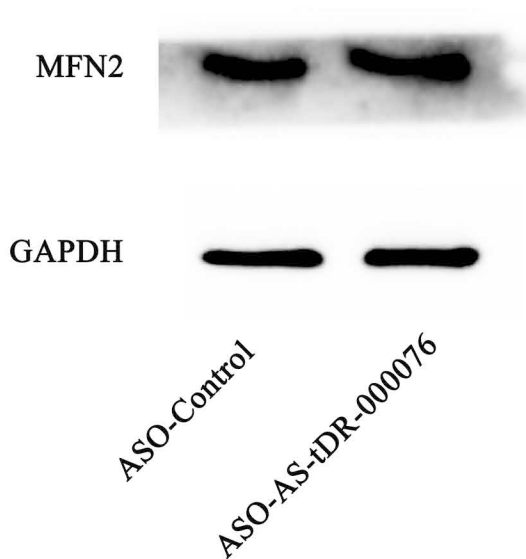

Supplement: Supplementary file 12 — Additional file 12: Figure S7. Original images for blots in Fig. 5. [file 11658_2022_346_MOESM12_ESM.pdf]

Original images for blots in Figure S1A.

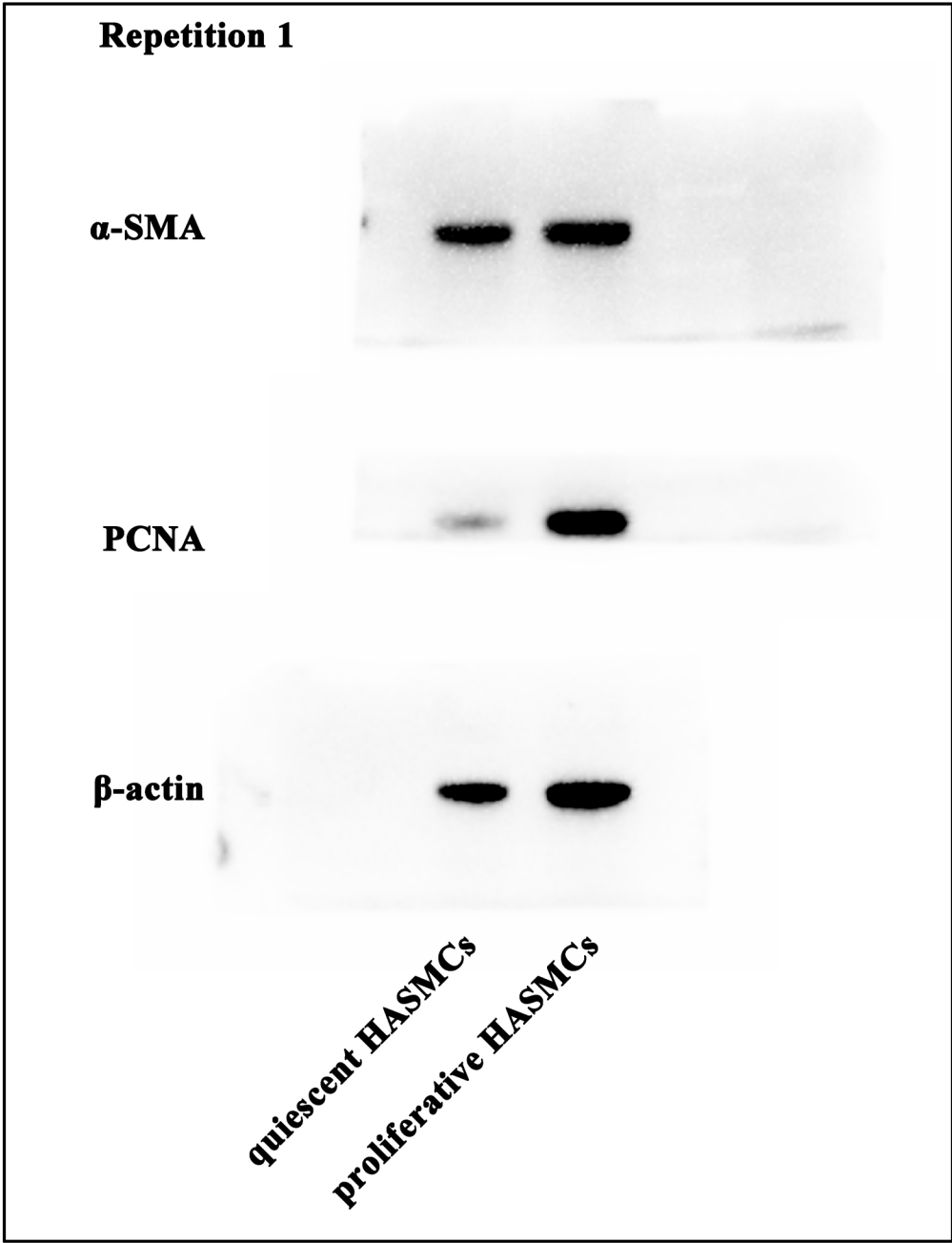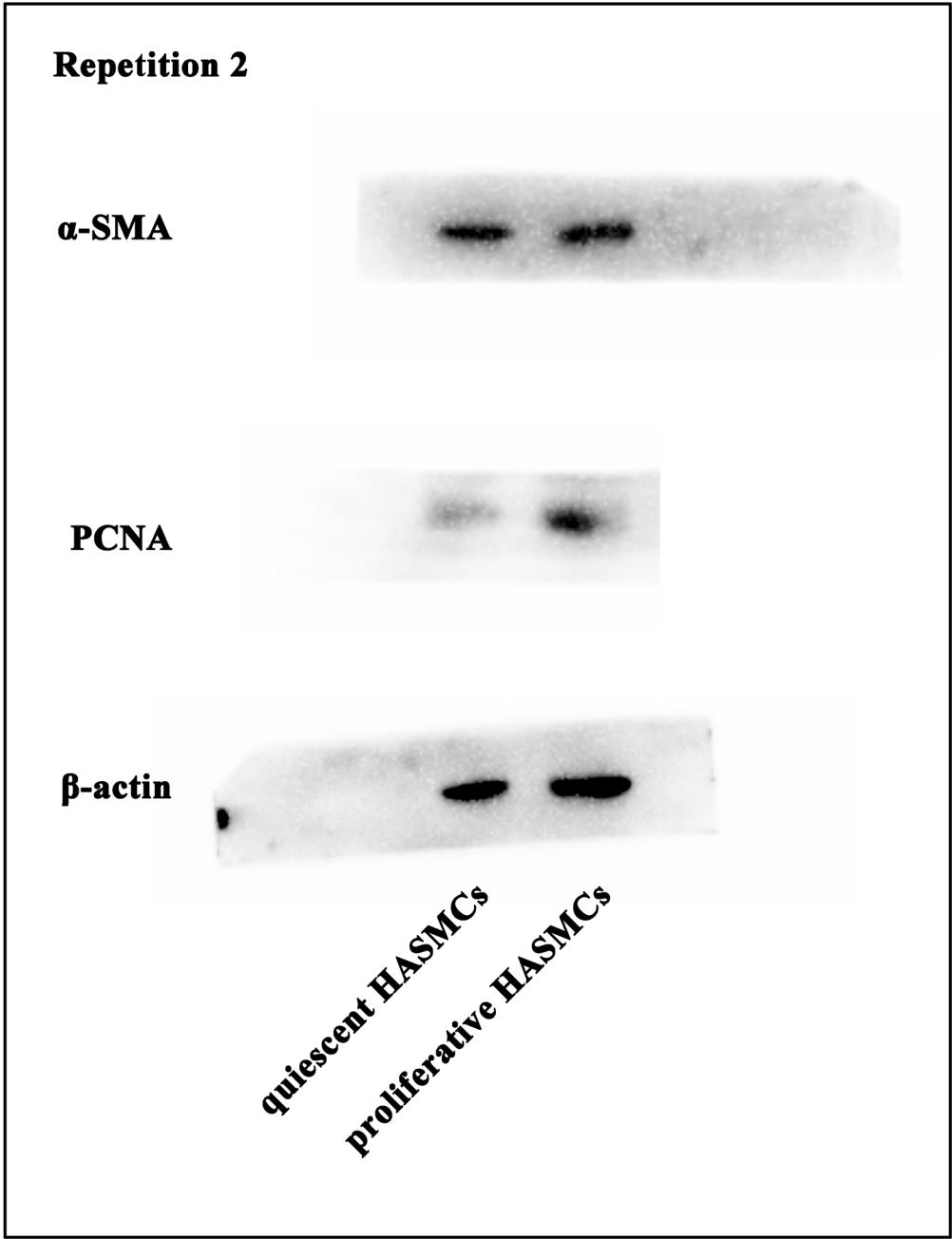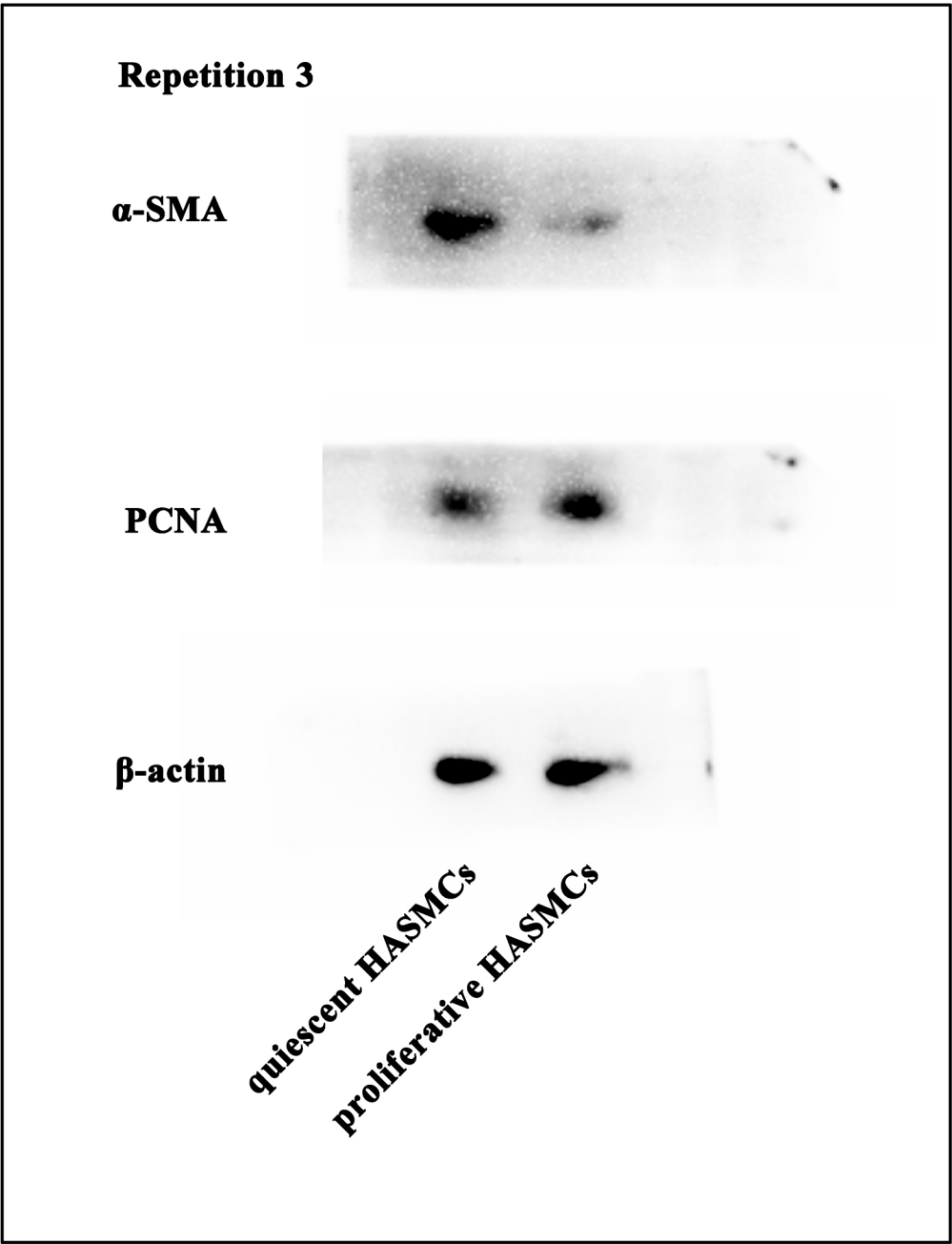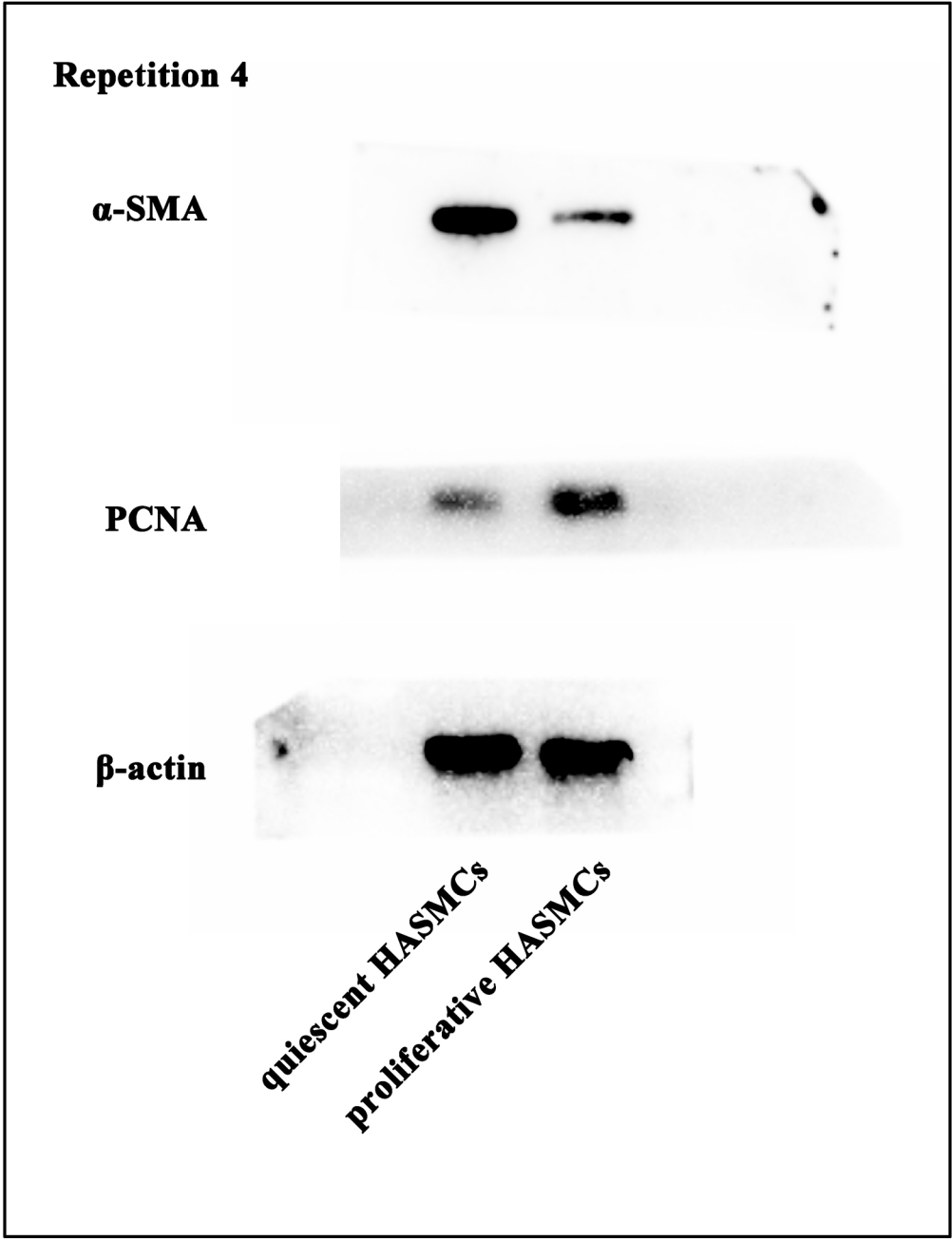

Supplement: Supplementary file 13 — Additional file 13: Figure S8. Original images for blots in Fig. S1A. [file 11658_2022_346_MOESM13_ESM.pdf]
